# A Drosophila model for toxicogenomics: Genetic variation in susceptibility to heavy metal exposure

**DOI:** 10.1371/journal.pgen.1006907

**Published:** 2017-07-21

**Authors:** Shanshan Zhou, Sarah E. Luoma, Genevieve E. St. Armour, Esha Thakkar, Trudy F. C. Mackay, Robert R. H. Anholt

**Affiliations:** 1 Program in Genetics, W. M. Keck Center for Behavioral Biology, and Department of Biological Sciences, North Carolina State University, Raleigh, North Carolina, United States of America; 2 Enloe Magnet High School, Raleigh, North Carolina, United States of America; The University of North Carolina at Chapel Hill, UNITED STATES

## Abstract

The genetic factors that give rise to variation in susceptibility to environmental toxins remain largely unexplored. Studies on genetic variation in susceptibility to environmental toxins are challenging in human populations, due to the variety of clinical symptoms and difficulty in determining which symptoms causally result from toxic exposure; uncontrolled environments, often with exposure to multiple toxicants; and difficulty in relating phenotypic effect size to toxic dose, especially when symptoms become manifest with a substantial time lag. *Drosophila melanogaster* is a powerful model that enables genome-wide studies for the identification of allelic variants that contribute to variation in susceptibility to environmental toxins, since the genetic background, environmental rearing conditions and toxic exposure can be precisely controlled. Here, we used extreme QTL mapping in an outbred population derived from the *D*. *melanogaster* Genetic Reference Panel to identify alleles associated with resistance to lead and/or cadmium, two ubiquitous environmental toxins that present serious health risks. We identified single nucleotide polymorphisms (SNPs) associated with variation in resistance to both heavy metals as well as SNPs associated with resistance specific to each of them. The effects of these SNPs were largely sex-specific. We applied mutational and RNAi analyses to 33 candidate genes and functionally validated 28 of them. We constructed networks of candidate genes as blueprints for orthologous networks of human genes. The latter not only provided functional contexts for known human targets of heavy metal toxicity, but also implicated novel candidate susceptibility genes. These studies validate Drosophila as a translational toxicogenomics gene discovery system.

## Author summary

Although physiological effects of environmental toxins are well documented, we know little about the genetic factors that determine individual variation in susceptibility to toxins. Such information is difficult to obtain in human populations due to heterogeneity in genetic background and environmental exposure, and the diversity of symptoms and time lag with which they appear after toxic exposure. Here, we show that the fruit fly, Drosophila, can serve as a powerful genetic model system to elucidate the genetic underpinnings that contribute to individual variation in resistance to toxicity, using lead and cadmium exposure as an experimental paradigm. We identified genes that harbor genetic variants that contribute to individual variation in resistance to heavy metal exposure. Furthermore, we constructed genetic networks on which we could superimpose human counterparts of Drosophila genes. We were able to place human genes previously implicated in heavy metal toxicity in biological context and identify novel targets for heavy metal toxicity. Thus, we demonstrate that based on evolutionary conservation of fundamental biological processes, we can use Drosophila as a powerful translational model for toxicogenomics studies.

## Introduction

Studies on the genetics of susceptibility to environmental toxins are challenging in human populations, due to the variety of clinical symptoms and difficulty in determining which symptoms causally result from toxic exposure; uncontrolled environments, often with exposure to multiple toxicants; and difficulty in relating phenotypic effect size to toxic dose, especially when symptoms become manifest with a substantial time lag after exposure. A variety of conventional model systems are used extensively for toxicological studies, including cell lines to assess the effects of toxicants on cellular processes [1, 2], zebrafish to evaluate adverse effects of toxicants on development [3], Daphnia as an ecological sentinel [4, 5], and rodents to evaluate physiological and behavioral effects of toxicants [6, 7]. However, the Drosophila model is eminently suitable as a model system for population-based large scale genomic studies that can explore genetic factors that underlie individual variation in susceptibility/resistance to toxicants.

*Drosophila melanogaster* is a powerful genetic model system for the identification of allelic variants that contribute to variation in resistance to environmental toxins in populations. To explore the Drosophila system as a translational model for toxicogenomic analyses, we took advantage of natural variants that segregate in the *D*. *melanogaster* Genetic Reference Panel (DGRP), a collection of 205 wild-derived sequenced inbred lines [8, 9], and focused on individual variation in sensitivity to heavy metal exposure.

Heavy metals are ubiquitous in the environment. Some heavy metals (e.g. zinc, copper, iron) are essential metabolic trace elements that serve as cofactors for enzymatic reactions, but are toxic when present in excess. Other heavy metals, including lead and cadmium, do not occur naturally in biological systems. The mechanisms of their toxicity are diverse and may include competition for endogenous enzymatic cofactors, effects on ion channels, or oxidative damage. Since these compounds readily cross the blood-brain barrier, they also affect the central nervous system. Homeostasis of essential metal trace elements is mediated by metallothioneins, small (5-7kD) cysteine rich proteins. Metallothioneins can also bind ingested toxic heavy metals, notably cadmium [10–12]. Exposure to toxic heavy metals results in the induction of metallothioneins as a protective physiological response [12–14]. Sequestration of heavy metals in bone and their binding to metallothioneins as well as albumin contributes to their long persistence with half-lives that can extend over many years [10, 11, 15].

Lead exposure is especially detrimental during early development and even low doses can result in intellectual disability [16–20] as well as behavioral disorders [21–24]. The neurotoxic effects of lead on the nervous system may be mediated through its effects on the function of hippocampal NMDA receptors and inhibition of presynaptic calcium channels [25, 26]. Lead compounds have been used in paints, and lead paint exposure is a major route for lead ingestion in children [27, 28]. Adult exposure to lead occurs in a variety of occupational settings [24] and can—among other effects—result in cognitive [29] and cardiovascular [30] disorders.

Cadmium is an exceptionally toxic heavy metal used extensively in electroplating as well as in the manufacture of some batteries, and is also found in certain fertilizers. The most extensively documented case of cadmium poisoning is the occurrence of itai-itai disease in Japan [31, 32]. Cadmium polluted irrigation water was used to irrigate rice fields, and subsequent consumption of rice resulted in symptoms characterized by fragmentation and deterioration of bone and compromised kidney function. The effects on the kidney are the result of glomerular filtration of the cadmium-metallothionein complex, which is subsequently reabsorbed in proximal tubules, where the metallothionein is degraded and free cadmium is released, resulting in proximal tubular cell damage through cadmium-induced oxidative stress [32–34]. Cadmium-induced proximal renal tubular dysfunction has deleterious effects on ion balance. Loss of calcium from bones and its excretion in the urine increases risk of kidney stones [32, 35]. In addition to its toxic effects on renal function, bone metabolism and cardiovascular function [34], cadmium has also been identified as a carcinogen [32, 33, 36, 37].

Although the clinical effects and pharmacodynamics of heavy metal toxicity have been extensively studied, little is known about the genetic factors that determine individual variation in sensitivity to toxic heavy metal exposure. A few human studies have examined associations of polymorphisms in candidate genes with lead [38, 39] or cadmium [40] blood concentrations; with maternal lead burden and infant birth weight [41]; and cadmium associated effects on bone mineral density [42]. However, genetic studies of sensitivity and resistance to heavy metal toxicity in human populations have often been inconclusive, mostly due to limited statistical power [43]. In addition, cadmium-induced histone modifications at the metallothionein MT3 promoter have been reported [44], and it has been suggested that changes in DNA methylation that may affect expression of DNA repair and tumor suppressor genes could mediate the carcinogenic effects of cadmium [45].

Previously, we performed a genome-wide association (GWA) analysis using the DGRP and identified polymorphisms associated with variation in sensitivity to lead toxicity by quantifying development time and viability [46]. Effects of lead exposure on adult locomotor activity have also been documented both by a QTL mapping study in recombinant inbred lines constructed from parental Oregon R and Russian 2b lines [47] and in the DGRP [46]. Here, we study the effects of heavy metals on adult survival by capitalizing on natural genetic and phenotypic variation in an outbred advanced intercross population (AIP) derived from a base population of 37 maximally homozygous and unrelated DGRP lines, free of chromosomal inversions and the endosymbiont *Wolbachia*. Following many generations of recombination, we tested survival of male and female adult flies following exposure to either lead or cadmium, and identified alleles with significant differences in allele frequencies between the top 10% most resistant individuals and a random sample of unexposed individuals using whole genome DNA sequencing (extreme QTL mapping) [48–50]. Since this scenario enables us to assay and pool unlimited numbers of unique genotypes, we increase statistical power compared to using a small number of DGRP lines. Furthermore, alleles that are present at low frequency (less than 5%) in the DGRP, and may have large phenotypic effects but cannot be detected by GWA analysis in the DGRP, are detectable in the extreme QTL mapping design using the AIP [48, 50]. Together, many segregating alleles with varying phenotypic effects and their interactions determine the extent of genetic sensitivity/resistance to heavy metal exposure for a given individual.

We identified SNPs associated with variation in resistance to both cadmium and lead, as well as SNPs associated with variation in resistance specific to one of these two heavy metals. The SNPs had largely sex-specific effects on resistance to both heavy metals. We constructed genetic interaction networks to place candidate genes tagged by the significant SNPs into biological context, and functionally assessed the candidate genes using mutant alleles and RNAi knockdown constructs. Finally, we were able to construct orthologous networks of human genes based on evolutionary conservation of fundamental cellular processes, some of which had been implicated previously with susceptibility to heavy metal exposure and many of which are novel candidate genes. These studies establish *D*. *melanogaster* as a powerful toxicogenomic model system.

## Results

### Extreme QTL mapping for lead and cadmium resistance

**Fig 1:**
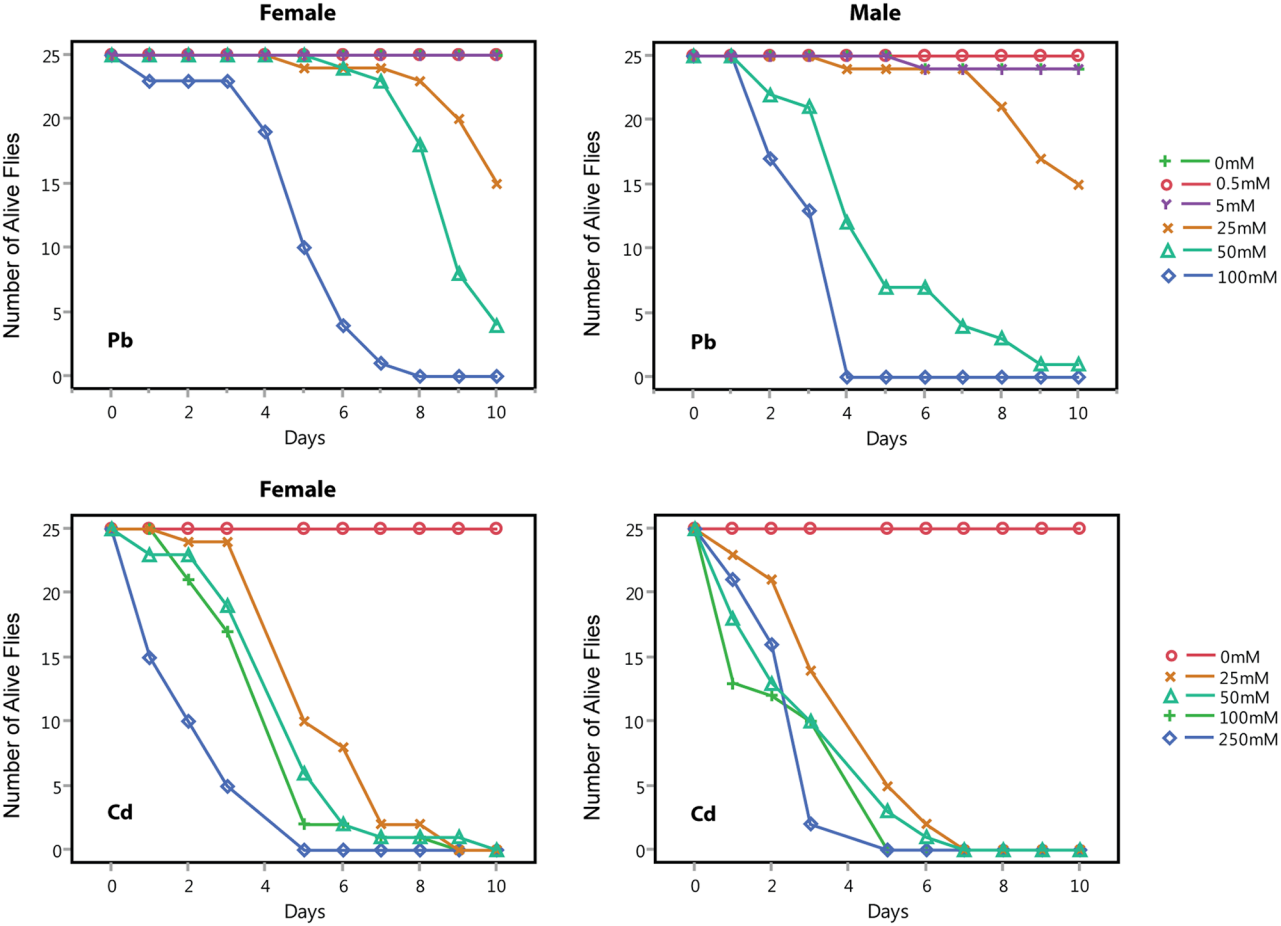
Dose-response curves for survival of adult AIP flies exposed to lead acetate (top panels) or cadmium chloride (bottom panels).

A previous study documented effects of rearing Canton-S flies on low concentrations of lead acetate (2–100μg/g) on courtship, fecundity and locomotor activity [51]. However, phenotypic characterization of the DGRP showed that variation in genetic background greatly affects susceptibility to lead exposure [46]. Therefore, we first established an optimal discriminating concentration of lead and cadmium to select individuals in our DGRP-derived outbred population, who would show extreme resistance to exposure to these heavy metals within a relatively short time span (about 7 days). This time window enables rapid high throughput screening, while ensuring that survival reflects heavy metal resistance rather than starvation resistance due to food avoidance. Dose-response survival curves showed that AIP flies are more sensitive to cadmium than to lead exposure (Fig 1). Exposure up to 5 mM lead acetate had little effect on survival during a 10-day assay period, whereas 100 mM lead acetate resulted in death of all females within 8 days and males within 4 days. We established 75 mM as an optimal concentration for identifying resistant individuals in the AIP for both sexes. Both sexes showed similar sensitivity to cadmium chloride with an optimal discriminating concentration of 25 mM (Fig 1).

**Fig 2:**
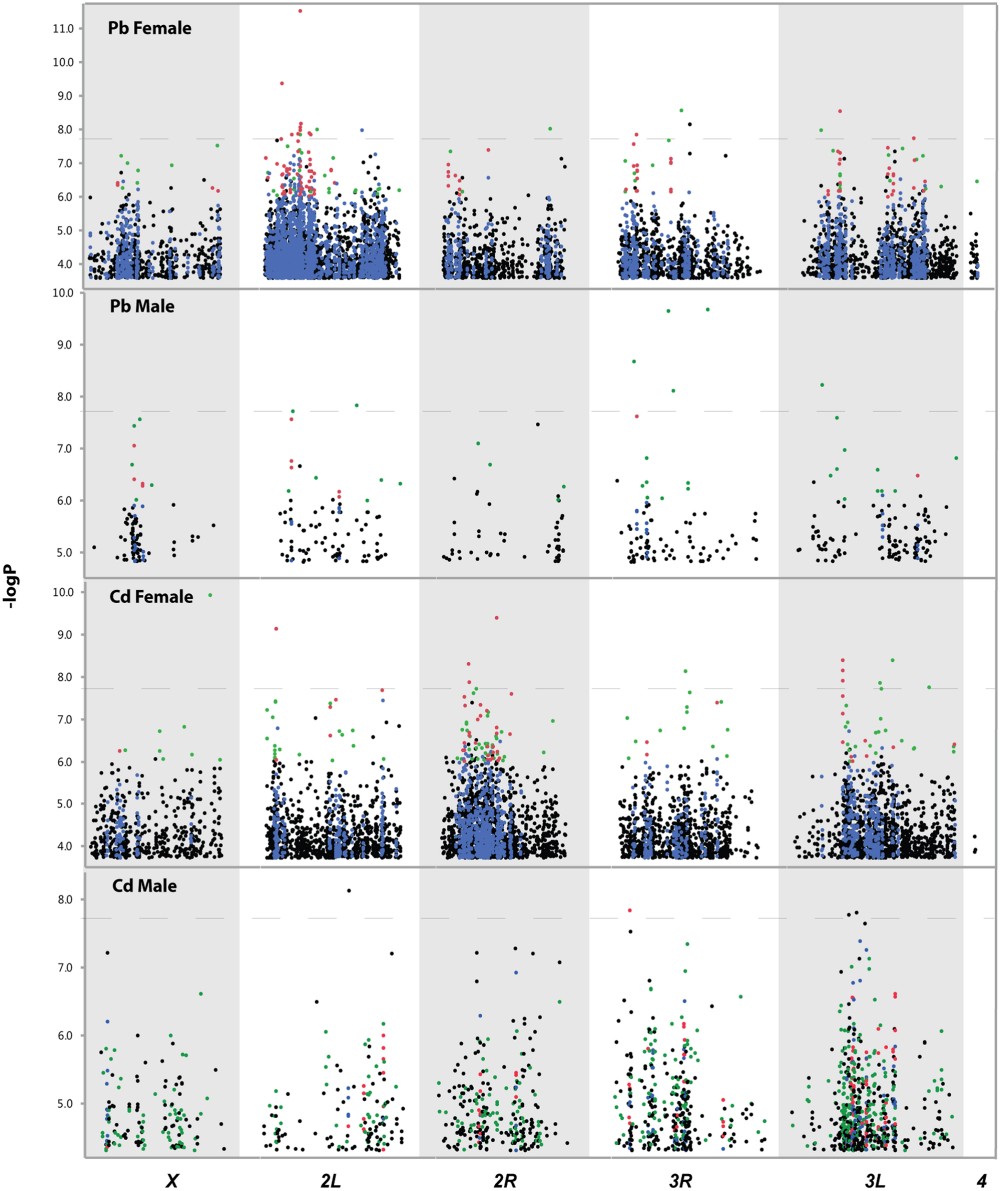
Extreme QTL mapping for variation in resistance to lead and cadmium exposure of adult flies. Manhattan plots show all SNPs significantly associated with resistance to lead or cadmium exposure at FDR<0.05. The *X*-axis designates the chromosomal arms of the Drosophila genome. SNPs are color coded. Blue dots indicate SNPs in or near a gene that harbors multiple significant SNPs (≥5), green dots indicate SNPs with large allele frequency differences between the resistant population and the control population (≥2 fold), and red dots indicate SNPs with large allele frequency differences in or near a gene that harbors multiple significant SNPs(≥5). The horizontal dashed lines indicate the Bonferroni-corrected threshold for statistical significance.

We collected the 10% surviving males and females reared on 75 mM lead acetate and 25 mM cadmium chloride as well as randomly selected unexposed control flies (*n* = 300 resistant and 300 control flies, pooled in three groups of 100 resistant or control flies). We performed whole genome DNA sequencing of the 24 pooled samples and identified alleles with significant differences in frequencies between the resistant and control samples, separately for each sex and the two heavy metal treatments (S1 Fig and Fig 2). At an FDR ≤ 0.05, we identified 8,190 differentially segregating SNPs in females, but only 465 in males (S1 Table), indicating that alleles with significant sex-specific effects underlie susceptibility to lead acetate (Fig 2). Similarly, for cadmium chloride exposure we identified 5,981 differentially segregating SNPs in females and far fewer, 1,555, in males (Fig 2; S2 Table). Whereas there was little or no overlap of SNPs between sexes and treatments, 188 genes were in common between males and females for lead exposure and 389 genes were in common between the sexes for exposure to cadmium. Furthermore, 51 genes were in common between lead and cadmium exposure in males and 1,035 were in common between the two treatments in females (S2 Fig). A total of 3,261 significant SNPs are located in intergenic regions (S1 and S2 Tables). A total of 2,520 genes tagged by significant SNPs encode transcripts of unknown function, 530 encode non-coding RNAs and 57 encode microRNAs (S1 and S2 Tables).

When we applied a more stringent Bonferroni threshold for statistical significance based on 2,636,680 SNPs tested (*P* < 1.896 x 10^−8^), we identified 20 SNPs in females (tagging 21 genes and three intergenic SNPs), and six in males (corresponding to five genes and one intergenic SNP) associated with resistance to lead acetate; and 13 SNPs in females (tagging 14 genes and two intergenic SNPs) and four in males (tagging five genes) associated with resistance to cadmium chloride (Fig 2; Table 1; S1 Fig). None of these SNPs result in nonsynonymous substitutions. The majority of significant SNPs occur in intronic regions or upstream or downstream of their corresponding genes, indicating that they are likely to exert their effects by regulating gene expression. Four SNPs associated with lead resistance in females and two SNPs associated with cadmium resistance in females are annotated to be associated with more than one gene. One SNP associated with cadmium resistance in males is associated with two genes, *Abi* and *twf* (Table 1). It is of interest to note that among all the genes associated with resistance to lead or cadmium at a Bonferroni-corrected level of significance, 21 are poorly annotated or encode transcripts of unknown function. Among the five candidate genes implicated in resistance to cadmium in males, three (*Tm1*, *Abi* and *twf*) are actin binding proteins involved in actin cytoskeleton organization; and one (*Dyrk2*) encodes a serine/threonine protein kinase, suggesting that integrity of the cytoskeleton might contribute to cadmium resistance, at least in males.

**Table 1:** SNPs and candidate genes associated with resistance to lead and cadmium exposure from extreme QTL analyses that passed the Bonferroni-corrected threshold (*P* < 1.896 x 10^−8^).

SNP ID	Flybase ID	Gene Symbol	Site Class	Metal	Sex
*2L*_16532374_SNP	FBgn0032587	*CG5953*	INTRON	Lead	Female
*2L*_2683318_SNP	FBgn0051690	*CG31690*	INTRON	Lead	Female
*2L*_2883366_SNP	FBgn0024947	*NTPase*	INTRON	Lead	Female
*2L*_4523837_SNP	FBgn0053196	*dp*	INTRON	Lead	Female
*2L*_5626473_SNP	FBgn0051646	*CG31646*	UTR_3_PRIME	Lead	Female
*2L*_5933037_SNP	FBgn0015381	*dsf*	INTRON	Lead	Female
*2L*_5933039_SNP	FBgn0015381	*dsf*	INTRON	Lead	Female
*2L*_5961505_SNP	Intergenic	*Intergenic*	Intergenic	Lead	Female
*2L*_5968844_SNP	FBgn0031757	*Ucp4C*	SYNONYMOUS_CODING	Lead	Female
*2L*_6051088_SNP	FBgn0031769	*CG9135*	INTRON	Lead	Female
*2L*_6051088_SNP	FBgn0031768	*CG12393*	DOWNSTREAM	Lead	Female
*2L*_7466454_SNP	FBgn0031918	*CG6055*	UPSTREAM	Lead	Female
*2L*_7703923_SNP	FBgn0041181	*Tep3*	INTRON	Lead	Female
*2L*_8863409_SNP	FBgn0052986	*CG32986*	SYNONYMOUS_CODING	Lead	Female
*2L*_8863409_SNP	FBgn0266798	*CR45260*	EXON	Lead	Female
*2L*_8863409_SNP	FBgn0052987	*CG32987*	UPSTREAM	Lead	Female
*2R*_18509894_SNP	FBgn0053143	*CG33143*	INTRON	Lead	Female
*3L*_10848282_SNP	Intergenic	*Intergenic*	Intergenic	Lead	Female
*3L*_12347519_SNP	Intergenic	*Intergenic*	Intergenic	Lead	Female
*3L*_3173545_SNP	FBgn0035410	*CG14964*	SYNONYMOUS_CODING	Lead	Female
*3L*_3173545_SNP	FBgn0035409	*CG14963*	UPSTREAM	Lead	Female
*3R*_20463412_SNP	FBgn0039214	*puf*	INTRON	Lead	Female
*3R*_4613931_SNP	FBgn0261015	*Pif1A*	INTRON	Lead	Female
*3R*_4613931_SNP	FBgn0046874	*Pif1B*	INTRON	Lead	Female
*3R*_7930140_SNP	FBgn0051361	*dpr17*	INTRON	Lead	Female
*3L*_15073689_SNP	FBgn0086690	*cp309*	INTRON	Lead	Male
*3L*_8871259_SNP	FBgn0263930	*dally*	INTRON	Lead	Male
*3L*_3311057_SNP	FBgn0035432	*ZnT63C*	INTRON	Lead	Male
*3R*_5303291_SNP	FBgn0037698	*CG16779*	INTRON	Lead	Male
*3L*_9656385_SNP	FBgn0016081	*fry*	INTRON	Lead	Male
*2L*_15289857_SNP	Intergenic	*Intergenic*	Intergenic	Lead	Male
*X*_20503276_SNP	Intergenic	*Intergenic*	Intergenic	Cadmium	Female
*2R*_9348948_SNP	FBgn0000119	*arr*	INTRON	Cadmium	Female
*2L*_1611464_SNP	FBgn0031351	*CG14352*	UTR_5_PRIME	Cadmium	Female
*2L*_1611464_SNP	FBgn0051935	*CG31935*	UPSTREAM	Cadmium	Female
*2L*_1611464_SNP	FBgn0021906	*RFeSP*	DOWNSTREAM	Cadmium	Female
*3R*_17067239_SNP	Intergenic	*Intergenic*	Intergenic	Cadmium	Female
*3R*_8564865_SNP	FBgn0038082	*CG5724*	UPSTREAM	Cadmium	Female
*2R*_4550989_SNP	FBgn0003892	*ptc*	INTRON	Cadmium	Female
*3L*_11632664_SNP	FBgn0052091	*CG32091*	INTRON	Cadmium	Female
*3R*_8564862_SNP	FBgn0038082	*CG5724*	UPSTREAM	Cadmium	Female
*2R*_4700759_SNP	FBgn0024189	*sns*	INTRON	Cadmium	Female
*3R*_14861896_SNP	FBgn0038676	*CG6026*	INTRON	Cadmium	Female
*3R*_8564866_SNP	FBgn0038082	*CG5724*	UPSTREAM	Cadmium	Female
*2R*_5844183_SNP	FBgn0011656	*Mef2*	INTRON	Cadmium	Female
*3R*_23372802_SNP	FBgn0046885	*Gr98d*	INTRON	Cadmium	Female
*3R*_23372802_SNP	FBgn0004387	*Klp98A*	DOWNSTREAM	Cadmium	Female
*2L*_14184469_SNP	FBgn0016930	*Dyrk2*	DOWNSTREAM	Cadmium	Male
*3L*_2869876_SNP	FBgn0263392	*CG43444*	INTRON	Cadmium	Male
*3R*_11127964_SNP	FBgn0003721	*Tm1*	INTRON	Cadmium	Male
*3R*_9948169_SNP	FBgn0020510	*Abi*	UPSTREAM	Cadmium	Male
*3R*_9948169_SNP	FBgn0038206	*twf*	UPSTREAM	Cadmium	Male

### Functional analysis of candidate susceptibility genes

We used mutants and RNAi-mediated suppression of gene expression to assess whether candidate genes that harbor SNPs associated with variation in resistance to lead or cadmium themselves affect heavy metal sensitivity. We obtained available *Mi{ET1}* mutants in the *w*^*1118*^ genetic background and *UAS-RNAi* lines without predicted off-target effects from the VDRC collection. We tested 15 *Mi{ET1}* lines and 30 RNAi lines targeting 33 candidate genes (S3 Table). These genes were either associated with resistance to both lead and cadmium in both sexes or had highly significant (*P* < 10^−8^) effects in any one condition. A total of 12 genes (*beat-IIIc*, *cdi*, *CG17193*, *CG31760*, *CG32091*, *CG42389*, *dpr8*, *mgl*, *Nlg4*, *Plp*, *Ptp61F*, *Shab*) were tested using both *Mi{ET1}* mutants and RNAi lines.

In order to determine the optimally discriminating concentration for exposure to lead acetate and cadmium chloride for the *Mi{ET1}* mutants and RNAi lines, we performed dose-response analyses using the three control lines. When exposed to cadmium, the control lines showed similar responses compared to the AIP. At a concentration of 25 mM cadmium chloride, approximately 80% of the flies died by day 5 of exposure (Fig 1 and S3 Fig). However, the mutant and RNAi control lines are more resistant to lead exposure compared to the AIP. At a concentration of 150 mM lead acetate, approximately 80% of the flies died by day 5 of exposure (Fig 1 and S3 Fig). Therefore, we used concentrations of 25 mM cadmium chloride and 150 mM lead acetate supplemented medium for our functional analyses.

**Fig 3:**
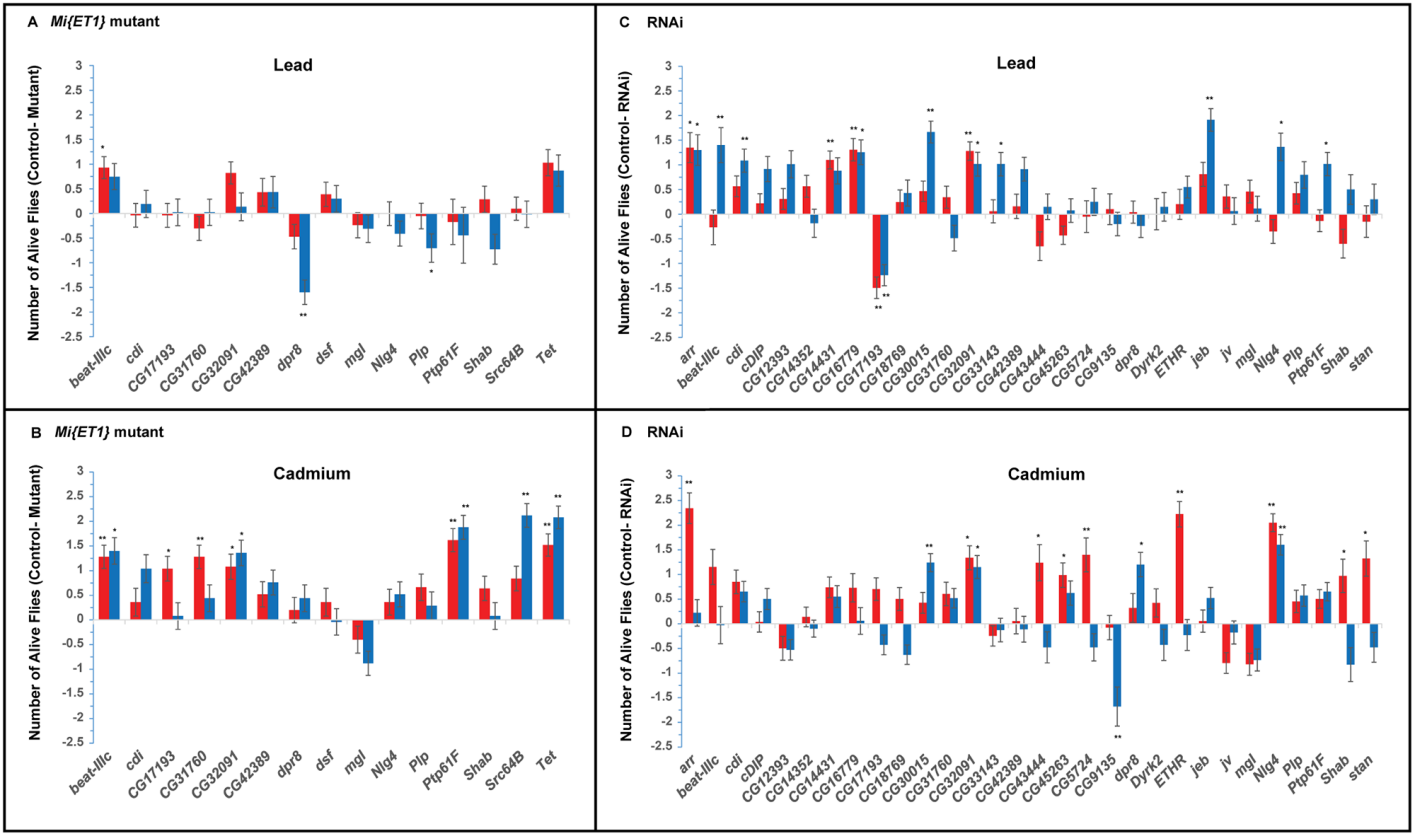
Functional analyses of candidate genes associated with resistance following lead and cadmium exposure of adult flies. The bar graphs represent differences between the number of flies alive for *Mi{ET1}* mutants (panels A and B) or RNAi lines (panels C and D) and their corresponding controls on the fifth day of exposure to lead (panels A and C) or cadmium (panels B and D). Bars above the horizontal line indicate increased susceptibility to heavy metal exposure, whereas bars below the horizontal line indicate increased resistance. Red bars indicate females and blue bars indicate males. Error bars denote standard errors. *P-*values are from the reduced ANOVA model. **: *P*<0.01, *: *P*<0.05.

We performed both full model and reduced model ANOVAs for each mutant or *UAS*-RNAi line with the corresponding control line to assess the effect of the mutation or RNAi-targeted suppression of expression on sensitivity to lead and/or cadmium. We found significant effects for 28 genes (84% of the tested genes) for at least one of the line terms (line, line by sex, line by treatment, line by sex by treatment) from the full model ANOVA and/or for the line term from the reduced model ANOVA (*P* < 0.05) (S4 and S5 Tables). Again, effects of the mutations and RNAi knockdown constructs were often sex-specific: *dpr8*, *beat*-*IIIc*, *cdi*, *CG17193*, *CG30015* and *jeb* affected susceptibility to lead exposure in males; while *CG14431*, *CG16779*, *CG17193* and *CG32091* affected sensitivity to lead exposure in females (Fig 3A and 3C). Similarly, *Ptp61F*, *Src64B*, *Tet*, *Cg30015* and *Nlg4* affected sensitivity to cadmium in males; while *beat*-*lllc*, *CG31760*, *Ptp61F*, *Tet*, *arr*, *Cg5724*, *ETHR*, and *Nlg4* affected sensitivity to cadmium exposure in females (Fig 3B and 3D). The majority of the mutant alleles and RNAi knockdown constructs have reduced survival when exposed to lead and cadmium compared to the controls (Fig 3), suggesting that the products of these genes are essential for survival when exposed to heavy metals. Interestingly, mutations and RNAi knockdown constructs of several genes actually resulted in increased survival on exposure to heavy metals when compared to the controls. It is not uncommon to observe different sexually dimorphic effects on resistance to lead or cadmium between the *Mi{ET1}* mutant or RNAi line affecting the same gene. Flies with mutations or RNAi suppression of gene expression of *CG17193* (both sexes) and *dpr8*, *Nlg4* and *Plp* (males) were more resistant to lead than the control; and flies with mutations or RNAi knockdown of *mgl* (both sexes), *CG9135* (males) and *jv* (females) were more resistant to cadmium than the control. Therefore, wild type expression of these gene products possibly limits survival following exposure to heavy metals.

### Gene ontology analyses and genetic networks

The genetic basis of natural variation in resistance to heavy metal exposure is clearly highly polygenic. Therefore, we performed Gene Ontology (GO) enrichment analyses to put the significant genes in biological context. In addition, we assessed to what extent these genes participated in previously curated genetic and physical interactions. GO analysis for all candidate genes associated with variation in resistance to lead and cadmium in both sexes indicates predominant enrichment for developmental genes, especially GO categories related to development of the nervous system (S6 Table). Neurodevelopmental gene enrichment is also evident when GO enrichment analyses are performed separately for resistance to lead (S6 Table) or resistance to cadmium (S6 Table), in both cases combining significant genes in males and females. A similar GO enrichment profile is observed when sexes are analyzed separately. Finally, we performed GO enrichment analyses for the subsets of genes in common between lead and cadmium, and between males and females (S7 and S8 Tables). Again, GO analyses indicated strong functional enrichment for neurodevelopment and connectivity (S8 Table).

**Fig 4:**
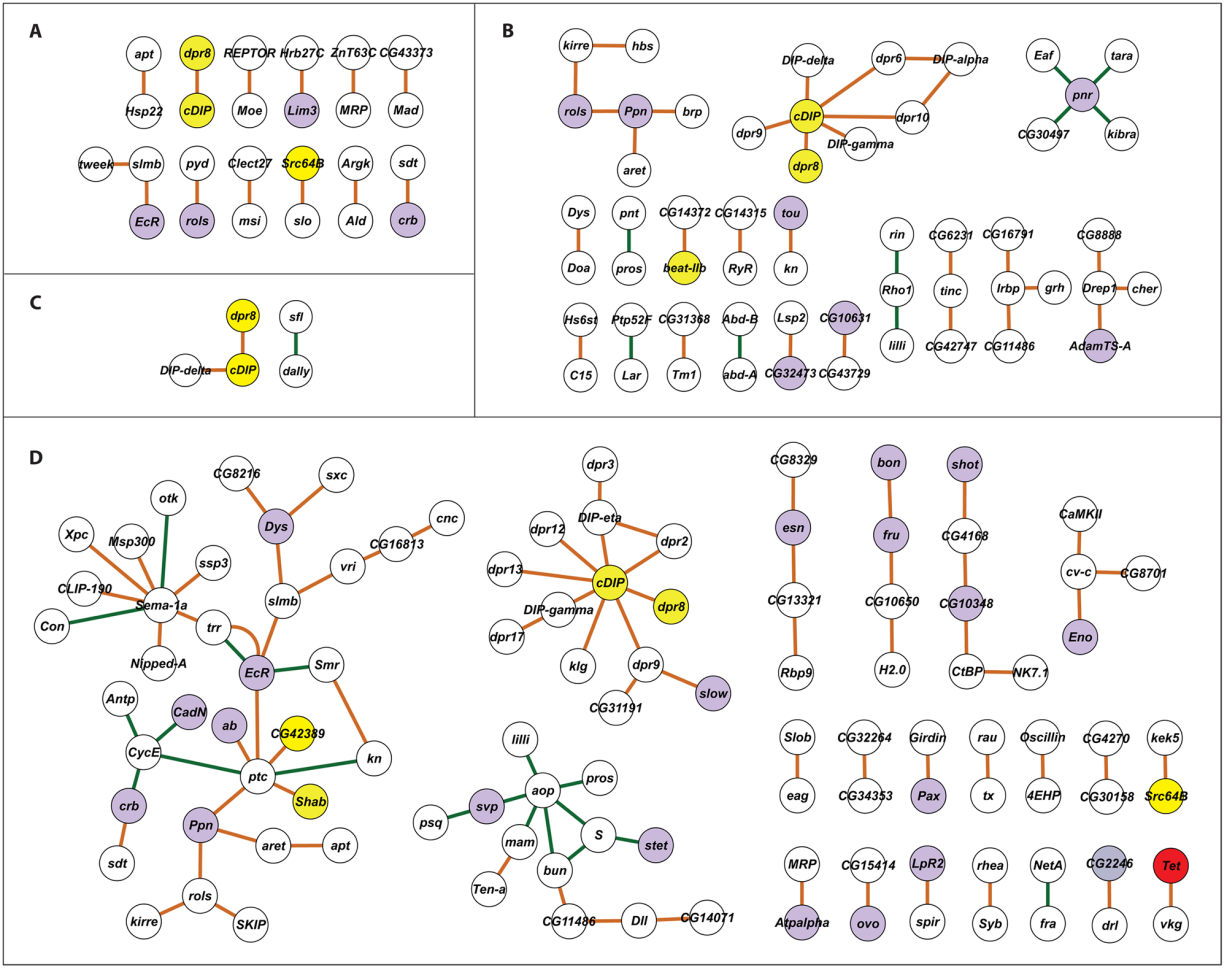
Networks associated with lead and cadmium susceptibility, based on known interactions between candidate genes in common among different exposure conditions. (A) Interactions between candidate genes associated with both female and male resistance to lead exposure. (B) Interactions between candidate genes associated with both female and male resistance to cadmium exposure. (C) Interactions between candidate genes associated with resistance to both lead and cadmium exposure in males. (D) Interactions between candidate genes associated with resistance to both lead and cadmium exposure in females. Orange edges indicate physical interactions and green edges indicate genetic interactions. Purple circles indicate genes annotated with divalent ion binding functions. Yellow circles indicate genes tested and functionally validated through mutational analysis. The red circle indicates a gene annotated with divalent ion binding function and validated through mutational analysis.

We next assessed to what extent the significant genes (*i*.*e*., genes in which one or more SNPs had an FDR < 0.05) that are shared between the sexes and/or between the heavy metal treatments are known to participate either in genetic and/or physical interactions. We searched for known genetic and physical interactions between our candidate genes using the esyN analysis portal [52]. The majority of the 188 genes in common between the sexes for lead resistance were not known to interact, except for one trio and 11 pairs of interacting genes (Fig 4A). The same was true for the 389 candidate genes associated with resistance to cadmium in both sexes, for which network analysis showed interactions only between 11 pairs, two trios, two sets of four connected genes, and one network each of five, six and eight genes (Fig 4B). Only 51 genes were in common for resistance to both lead and cadmium in males, of which only five were known to interact: a trio of genes (*dpr8*, *cDIP*, *DIP-delta*) and a single interacting pair (*dally* and *sfl*) (Fig 4C). In contrast, 1,035 genes are in common for resistance to lead and cadmium in females, and we found many more known interactions. Here, we identified a large interaction network consisting of 34 genes, two networks containing 13 genes, one trio, two groups of four genes, one network of five genes and 14 separate pairs (Fig 4D). The esyN analysis portal is limited in that only 700 input genes are allowed; therefore, we applied a threshold of FDR < 0.04 in this case to reduce the number of input genes for network analysis.

Members of the *dpr* gene family appear in all networks. Their gene products contain three immunoglobulin domains and belong to the immunoglobulin superfamily. They interact with Dpr-interacting proteins (DIPs) and act as neuronal surface markers that mediate specificity of synaptic connections. In addition, 21 candidate genes among the 164 genes included in the networks are transcriptional regulators, six of which contain zinc fingers (*EcR*, *fru*, *ovo*, *pnr*, *svp*, *tou*). Notably, 28 candidate genes from the networks in Fig 4 encode divalent ion binding gene products (Mg^2+^, Ca^2+^ or Zn^2+^), which are potential targets for interference by lead or cadmium. Among the 33 genes that we functionally assessed, seven are present in the networks shown in Fig 4, and all of them affected survival on exposure to heavy metals (Fig 3).

**Fig 5:**
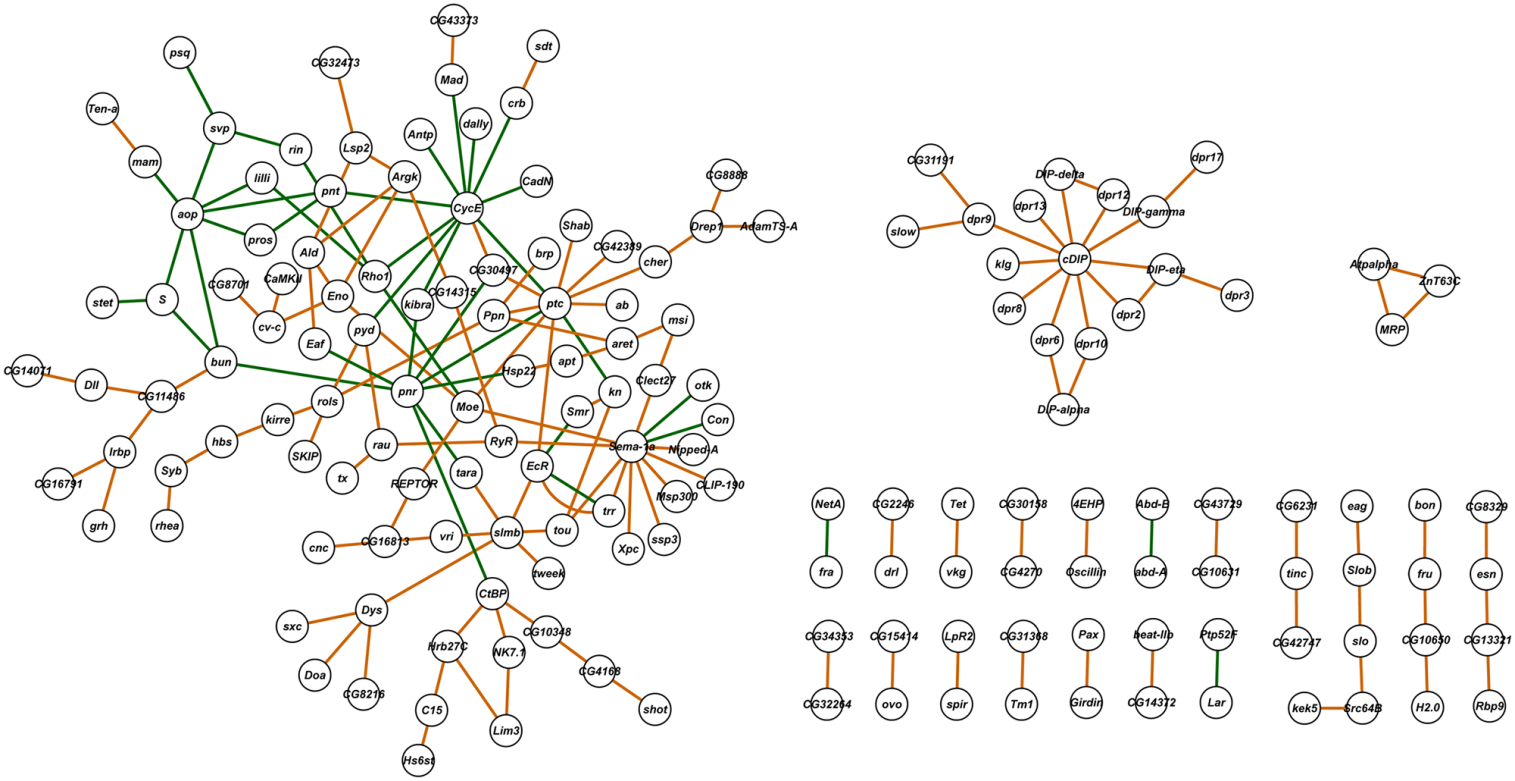
Networks derived from all candidate genes associated with heavy metal resistance that were in common between males and females or between lead and cadmium. Orange edges indicate physical interactions and green edges indicate genetic interactions.

It should be noted that our ability to resolve connectivity among the candidate genes is limited by the FDR values applied to declare significance of association, the limit on the number of input genes that can be entered into the esyN analysis portal, and prior knowledge of genetic and physical interactions among candidate genes, many of which include genes that encode predicted transcripts of unknown function. When all 164 genes that contribute to the networks in Fig 4 are combined in a single analysis, a well-integrated comprehensive network emerges (Fig 5). We identified *aop*, *CycE*, *pnr*, *ptc*, *Sema*-*1a*, *slmb*, *Moe* and *cDIP* as hub genes, since they interact with at least five genes in the network (Fig 5). *cDIP* encodes a product of unknown function, while the other hub genes include transcription factors and genes, which encode zinc ion binding proteins; they are involved with regulation of cell division, neurogenesis and cardioblast differentiation.

We also constructed networks for candidate genes uniquely implicated in only one sex and for only lead or cadmium exposure. However, these networks did not show significant enrichment of gene ontology categories.

### Translational networks: From flies to humans

Among the *D*. *melanogaster* candidate genes associated with resistance to lead or cadmium (S1 and S2 Tables), 3,348 (~59%) have human orthologs. These orthologs were also enriched in GO categories related to neural development. However, transport categories, including cation transport, featured prominently (S9 Table). Human orthologs of Drosophila genes associated with resistance only to cadmium in females were also enriched for functions of inactivation of MAPK activity, organic acid transmembrane transport, regulation of protein serine/threonine kinase activity, chromatin organization, renal water homeostasis and regulation of phosphate metabolism (S9 Table), whereas orthologs of genes associated with resistance exclusively to cadmium in males were also enriched for functions of organic acid transport, glutathione metabolism, lipid localization and sulfur compound metabolism (S9 Table). Furthermore, human orthologs of Drosophila genes uniquely associated with resistance to lead in females were also enriched for functions of flavonoid metabolism, carbohydrate metabolism, water homeostasis, SMAD protein signal transduction, regulation of peptidase activity and hemostasis (S9 Table), while those uniquely associated with resistance to lead in males were enriched for the GO category of peptide catabolism (S9 Table).

We identified human orthologs for each of the four common groups of Drosophila candidate genes, genes associated with lead and cadmium in females, genes associated with lead and cadmium in males, genes associated with lead in both females and males, and genes associated with cadmium in both females and males. All groups of human orthologs were enriched for functions of nervous system development, signaling and ion transport (S10 Table). In addition, orthologs of candidate genes associated with resistance to both lead and cadmium in females are enriched for GO categories of glycosylation and lipid transport.

In contrast to networks of Drosophila candidate genes that were common across sexes or treatments, networks of their corresponding human orthologs were larger, except for orthologs corresponding to Drosophila genes associated with susceptibility to both lead and cadmium exposures in males. Here, only two single interacting pairs emerged (*BOC* and *CDON*, and *DST* and *CELSR3*) (S4 Fig). In the orthologous network that is associated with exposure to both lead and cadmium in females we identified ten hub genes, *CFTR*, *CTBP1*, *DLG4*, *ENO1*, *NCOR1*, *NCOR2*, *PRKACA*, *PTPRK*, *RYK* and *VDR*. These genes have connections with more than ten other genes in the network. Furthermore, we identified two hub genes, *FLNA* and *FN1*, which are associated with cadmium resistance in both sexes. The orthologous network comprised of genes associated with exposure to cadmium for both sexes contained four hub genes, *SMAD9*, *TJP1*, *RYK* and *CFTR*. These hub genes were connected with more than five genes (S4 Fig, Table 2).

**Table 2:** Biological function annotations of hub genes from human orthologous networks.

Hub Gene	Function Summary
*BTRC*	Proteolysis
*CFTR*	regulation of chloride channel activity, cholesterol transport
*CTBP1*	regulation of histone acetylation
*DLG4*	regulation of cytosolic calcium concentration, neuronal synaptic plasticity
*ELAVL1*	regulation of stem cell population maintenance, RNA binding
*ENO1*	Glycolysis
*FBXW11*	Proteolysis
*FLNA*	actin skeleton, glycoprotein binding
*FN1*	calcium-dependent cell-matrix adhesion
*FYN*	metal ion binding, regulation of cell survival
*HSPB1*	heat shock protein, stress resistance, actin organization
*HSPB2*	heat shock protein, stress resistance, actin organization
*NCOR1*	regulation of lipid transport, chromatin binding
*NCOR2*	regulation of lipid transport, chromatin binding
*PRKACA*	calcium mediated signaling
*PTPRK*	protein localized to cell surface, regulation of cell adhesion, EGFR pathway
*RYK*	neuron differentiation, protein kinase
*SMAD9*	metal ion binding, bone and brain development
*SRC*	immune response, regulation of cell adhesion and apoptosis, ion channel binding
*TJP1*	calmodulin binding, cell-cell junction organization
*VDR*	calcium ion transport

**Fig 6:**
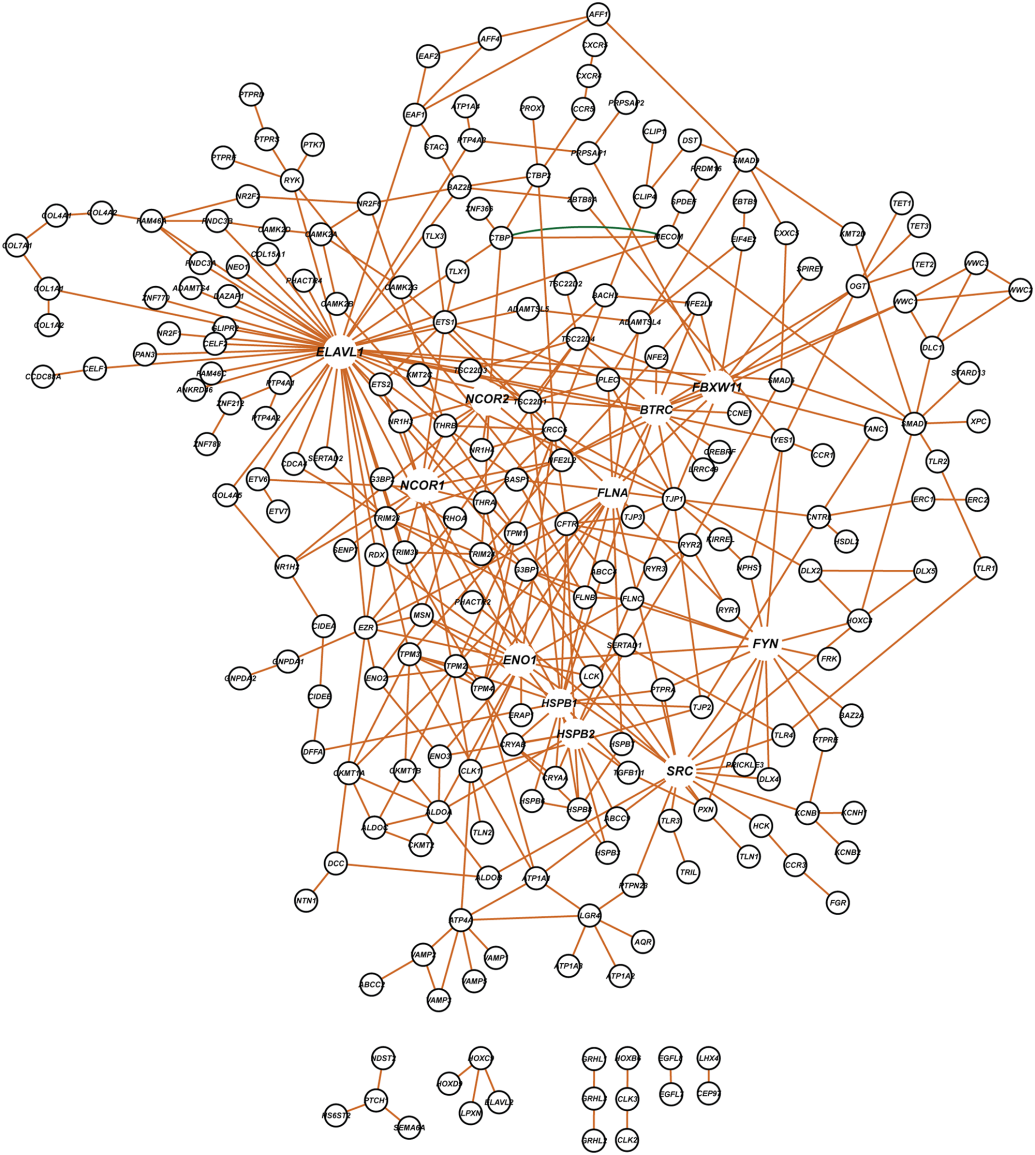
Network of human candidate genes orthologous to the Drosophila candidate genes shown in Fig 5. Orange edges indicate physical interactions and the green edge indicates a genetic interaction.

Finally, we constructed an orthologous human gene interaction network containing 148 genes (Fig 6) based on the comprehensive *D*. *melanogaster* network of Fig 5. There are instances where a single Drosophila gene corresponds to multiple human orthologs. We identified additional hub genes in this network, including *FBXW11*, *BRRC*, *FYN*, *HSPB1*, *HSPB2*, *SRC* and *ELAVL1*.

Among all the hub genes we identified in our analyses (Fig 6; S4 Fig) five encode calcium binding proteins, three affect chromatin structure and modification, two are involved in glycolysis, two are involved in proteolysis and eight genes regulate cytoskeletal structure that affects cell adhesion, cell-cell signaling and cell survival and proliferation (Table 2).

**Fig 7:**
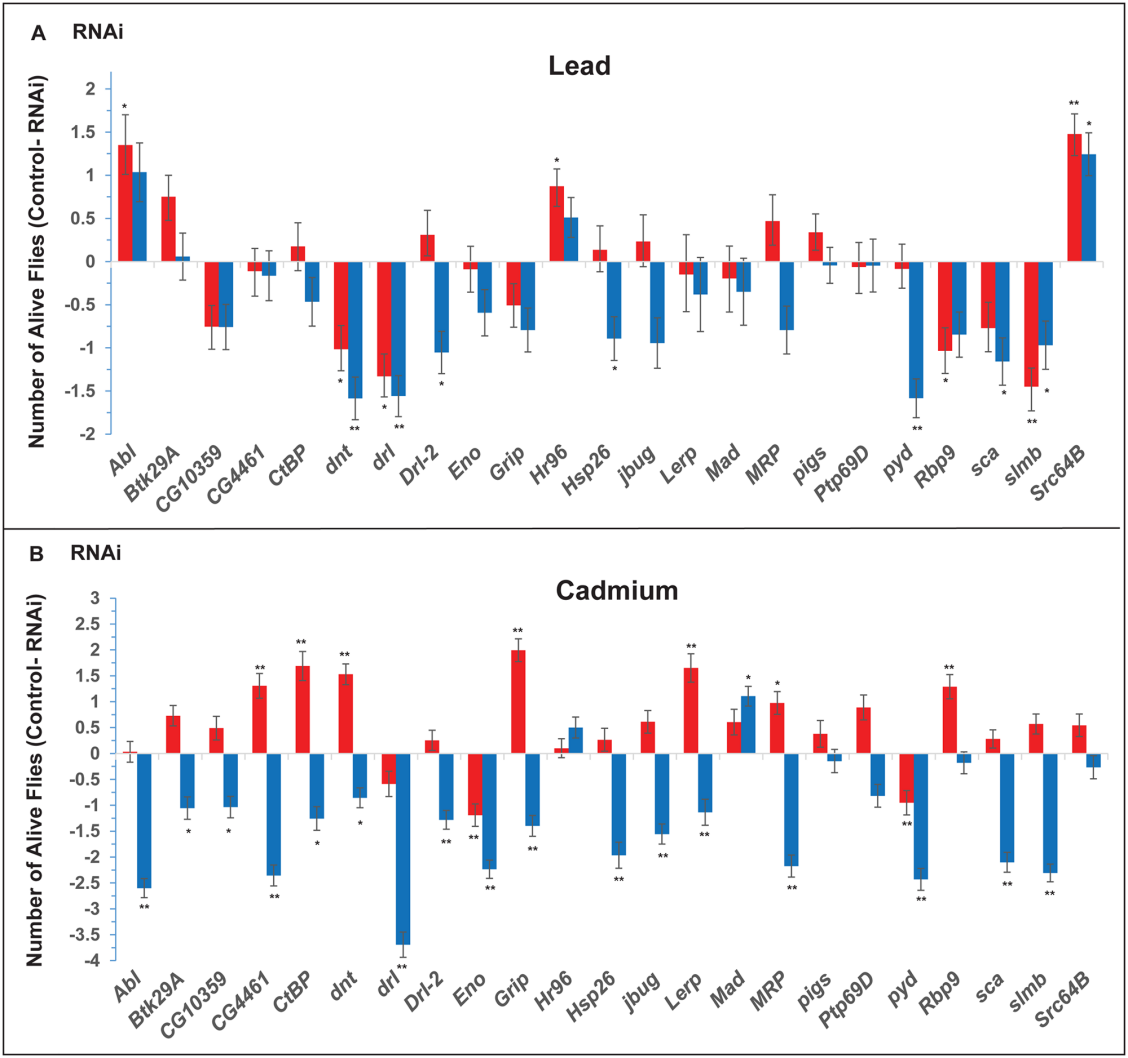
Functional analyses of candidate hub genes with human orthologs associated with resistance to lead and cadmium exposure of adult flies using RNAi targeted knockdown of gene expression. The bar graphs represent differences between the number of flies alive for RNAi lines and their corresponding controls on the seventh day of exposure to lead (A) or on the fifth day of exposure to cadmium (B). Bars above the horizontal line indicate increased susceptibility to heavy metal exposure, whereas bars below the horizontal line indicate increased resistance. Red bars indicate females and blue bars indicate males. Error bars denote standard errors. *P*-values are from the reduced ANOVA model. **: *P*<0.01, *: *P*<0.05. See also S11 Table.

We used targeted RNAi knockdown to functionally validate 23 Drosophila orthologs of human hub genes (Fig 7, S11 Table). We observed effects on susceptibility to lead or cadmium compared to controls in at least one sex for 20 (87%) of these lines. In many instances, RNAi knockdown rendered flies more resistant to heavy metal exposure, indicating that the candidate gene confers susceptibility. Fewer RNAi lines showed differences from control for exposure to lead (Fig 7A) than cadmium (Fig 7B). A surprising observation was that RNAi knockdown amplified sex differences when flies were exposed to cadmium, in most cases males becoming more resistant to cadmium exposure and females more susceptible (Fig 7B). RNAi knockdown of *Abl*, *dnt*, *drl*, *Drl-2*, *Hsp26*, *pyd*, *Rbp9*, *sca*, and *slmb* affected susceptibility/resistance to both heavy metals in at least one sex, often in opposite directions between exposure to lead and cadmium. Disruption of expression of *Hr96* and *Src64B* shows statistically significant differences from control specifically for exposure to lead (Fig 7A), while disruption of expression of *Btk29A*, *CG10359*, *CG4461*, *CtBP*, *Eno*, *Grip*, *jbug*, *Lerp*, *Mad* and *MRP* shows significant differences from control only for exposure to cadmium (Fig 7B).

To assess to what extent RNAi targeting reduced the mRNA of the target gene, we performed quantitative RT-PCR on a sample of 12 RNAi mutants and the control in males and females separately. With the weak ubiquitin driver used in these experiments we observed extensive variation among the extent of knockdown of the target gene ranging from 0 to 90% with average knockdown of 50% in males and 49% in females (S5 Fig). There was also sexually dimorphic variation in the extent of reduction in target mRNA. In most cases, we find no correlation between the extent of RNAi knockdown and phenotypic effect, *i*.*e*. a small reduction in expression of a specific gene may elicit a large phenotypic effect and *vice versa*.

## Discussion

### Extreme QTL mapping: A powerful genetic design

Although studies using conventional model systems, such as cell lines [1, 2], zebrafish [3], Daphnia [4, 5], mice and rats [6, 7], can provide important information about the cellular, developmental, physiological and behavioral effects of toxicants, these systems are not ideally suited to investigate the relationship between genetic variation and phenotypic variation in individual susceptibility to toxic exposure. Here, we show that Drosophila can serve as a powerful translational model for studies on the genetic basis of susceptibility to toxic exposure using resistance to heavy metal toxicity as an experimental paradigm.

In order to identify variants associated with resistance to lead and cadmium exposure in *D*. *melanogaster*, we implemented an extreme QTL mapping design [48, 49, 53] in which we compared allele frequencies of three replicate samples of randomly selected flies and three replicate samples of the 10% most resistant flies from an outbred population derived from 37 DGRP lines. We performed these experiments for each of the two heavy metals and separately for males and females. The outbred population segregates for 46% of the variants present in the 205 DGRP inbred lines. However, the extreme QTL mapping design has several advantages over a GWA study using the DGRP. First, the sample size is much larger, greatly increasing the power to detect associated variants. Second, the flies are outbred and do not suffer inbreeding depression. Third, extensive recombination during more than 60 generations of outcrossing among the AIP founder lines greatly increases the number of distinct genotypes. And finally, any rare alleles that are private to any of the founder lines were initially at a frequency of 2.7%. These alleles may have larger phenotypic effects than common alleles [8] and cannot be evaluated by single-marker GWA in the DGRP, but their effects can be assessed using this design.

### SNPs and networks

We identified SNPs associated with variation in resistance to both cadmium and lead, suggesting common cellular targets for the toxic effects of both heavy metals. In addition, we identified SNPs associated with variation in resistance specific to each of these heavy metals. Furthermore, we found striking sex-specific effects of associated variants, with more SNPs associated with variation to either cadmium or lead identified in females than in males, indicating that risk alleles for susceptibility may vary between the sexes, an observation that is likely to be relevant across phyla, including humans [54].

The cellular mechanisms by which SNPs give rise to variation in sensitivity to heavy metal exposure remain to be investigated. SNPs in promoter/enhancer regions may modulate gene expression levels, whereas SNPs in introns may affect alternative splicing and the conformation and stability of mRNA. Future studies in which genome-wide transcript abundance levels are correlated with DNA variants may help clarify the mechanisms by which allelic variants exert their effects on organismal phenotype.

Approximately 60% of the *D*. *melanogaster* candidate genes identified in our study have human orthologs. This enabled us to construct orthologous networks that identify candidate human hub genes associated with variation in heavy metal resistance and provide functional context. Many of the SNPs we identified are located in neurodevelopmental genes, suggesting that variation in nervous system connectivity may contribute to variation in survival upon exposure to high concentrations of lead or cadmium. SNPs associated with variation in survival when exposed to heavy metals may be different from those associated with variation in behavioral phenotypes observed at low concentrations of heavy metal exposure [51,55]. However, our observations are in line with a previous study on recombinant inbred lines, which used expression microarrays to identify *cis*-eQTL and *trans*-eQTL that were differentially expressed among control and lead-exposed flies. This study identified a co-regulated ensemble of 33 lead-induced genes many of which are associated with neurodevelopment [55].

A previous study showed that epistatic interactions between co-isogenic *P*-element insertion mutants that affect olfactory behavior undergo dynamic shifts when behavioral responses are measured at different odorant concentrations [56]. Thus, it is possible that the networks we have identified here may also show plasticity at different concentrations of heavy metal exposure, although hub genes are likely to be robust.

### From flies to humans

All genes tagged by significant SNPs are interesting candidate genes for future analyses. However, the advantage of utilizing natural variation is that we can gain insights about how combinations of significant genes act together to affect quantitative trait phenotypes. While many of these interactions are likely to be novel, we can utilize knowledge of genetic and physical interactions from the literature to develop heavy metal resistance-specific interaction networks. We identified elements of known genetic and/or physical interaction networks from genes that harbored significant SNPs that were in common between males and females in the analyses of the genetic basis of resistance to lead or cadmium, and genes that were in common between resistance to lead and cadmium in males and females. Combining all of these genes yielded a much larger integrated interaction network which places the candidate genes into functional context. Using mutants and RNAi knockdown of gene expression we functionally confirmed that 84% of the tested candidate genes indeed affected survival following exposure to lead and/or cadmium in at least one sex, similar to validation rates reported previously using GWA studies in the DGRP [46, 57–59]. Our functional analysis also showed that reducing expression of seven genes individually was sufficient to increase resistance to either lead or cadmium in one or both sexes.

We also used RNAi interference to functionally validate 20 of 23 Drosophila orthologs of hub genes in the analogous human genetic interaction network (Fig 6). The extent of RNAi knockdown was mostly not directly correlated with the phenotypic effect. A small reduction in expression of a specific gene may elicit a large phenotypic effect and *vice versa*. Thus, the effect of RNAi knockdown occurs within the context of a complex highly interconnected sexually dimorphic genetic architecture, which does not allow simple extrapolations to predict the extent and direction of the effect on the organismal phenotype.

We compared our results with those from previous studies on cadmium and lead toxicity in human populations or cell lines, and found that several genes identified by extreme QTL mapping in the Drosophila AIP correspond to previously identified human target genes. For example, polymorphisms in *VDR* have been implicated in sensitivity to lead toxicity [60, 61], and *VDR* also emerged as a hub gene from our network analyses (Table 2 and S4 Fig). *VDR* encodes the vitamin D3 receptor, which is essential for the metabolism of calcium and its incorporation in bone [62]. The Drosophila ortholog of *VDR*, *Hr3* (also known as *Hr46*), was associated with lead resistance in females as well as cadmium resistance in both sexes. Similarly, polymorphisms in human *HFE* have been associated with variation in heavy metal toxicity [63–66]. Human *HFE* does not have a Drosophila ortholog; however, orthologs of three of its interacting partners (*TF*, *HSPA5* and *SYVN1*), *Tsf3*, *Hsc70-2* and *sip3* were associated with either variation in cadmium resistance in females or variation in resistance to both lead and cadmium in females.

### Analysis of candidate resistance genes implicates oxidative stress

Oxidative stress is one mechanism by which exposure to lead gives rise to toxicity in humans. Lead exposure generates reactive oxygen species and depletes antioxidant reserves [67]. One of the primary pathways for protection against oxidative stress is mediated through glutathione [68]. We found that both *GstZ2* and *GstT3* harbored polymorphisms associated with variation in lead resistance in females. In addition, six additional *Gst* family members were associated with resistance to cadmium. Several enzymes, which play key roles in catalyzing oxidative reactions, are also targets of lead toxicity [67, 69]. The Drosophila genes *Trxr*-1, which encodes glutathione reductase; *PHGPx*, which encodes glutathione peroxidase; and *Sod* and *Sod3* encoding superoxide dismutase were all associated with lead resistance in females. Further, human orthologs of genes associated with variation in lead resistance in females included flavonoid metabolic genes, involved in protection against reactive oxygen species [70].

### Comparative aspects of lead toxicity

Recent studies show that epigenetic mechanisms play a role in lead toxicity. In human embryonic stem cells, decreased expression of *PAX6* and *MSl1* was coincident with an increase in DNA methylation upon exposure to lead [71]. We found that the Drosophila orthologs of these genes, respectively *toy* and *Rbp6*, were associated with lead resistance in both sexes and cadmium resistance in females. The Cincinnati Lead Study found that blood lead concentrations in childhood were associated with decreased DNA methylation of *PEG3* and *IGF2* [72]. The Drosophila orthologs of *PEG3*, *CG10431* and *CG7368* were associated with lead resistance in females, as well as cadmium resistance in both sexes. The Drosophila orthologs of *IGF2*, *Ilp1*, *Ilp5* and *Ilp7* were associated with lead resistance in females and cadmium resistance in males.

### Comparative aspects of cadmium toxicity

Like lead, cadmium also interferes with essential ions and accumulates in different tissues. Cadmium has been implicated in oxidative stress and as a carcinogen [32,73,74]. Human metallothionein binds cadmium and offers protection against cadmium toxicity, but cadmium-metallothionein complexes have been implicated in renal toxicity [75]. We found that polymorphisms in Drosophila *MtnB* and *MtnC*, which encode metallothioneins, were associated with variation in resistance to both cadmium and lead in females. Analyses of genome-wide transcriptional changes in human renal epithelial cells upon exposure to different concentrations of cadmium revealed a genetic network consisting of eight genes [76], four of which have Drosophila orthologs. SNPs in these orthologs—*daw* and *actbeta* (*INHBA*), *Droj2* (*DNAJA4*), *Hk*, *CG6084* and *CG6083* (*AKR1B10*) and *Hk* and *CG10683* (*AKR1C1*)—were associated with variation in resistance to cadmium and lead in females. Finally, studies on human cell lines reported associations between cadmium levels and expression levels of *HSD11B2*, *HIST1H4C* and *SATB2* in immortalized trophoblasts [77], HK*-*2 proximal tubular cells [78], and pancreatic ductal epithelial cells [79], respectively. Again, the respective corresponding Drosophila orthologs, *CG9265*, *His4r* and *dve* were associated with variation in resistance to cadmium in either females or males.

### Common cellular targets for lead and cadmium toxicity

Our study revealed common cellular pathways that may be affected by both lead and cadmium. One common mechanism points at disruption of intercellular signaling and cytoskeletal structure with resulting changes in cell adhesion, which plays a major role in regulating growth, differentiation and cell migration. Disruption of cell adhesion has also been implicated in the carcinogenic effects of heavy metals [80–82]. We also identified stress response genes, especially oxidative stress genes, to be associated with variation in either lead or cadmium resistance. In addition, association of intergenic SNPs, non-coding RNAs and microRNAs with variation in sensitivity to heavy metal toxicity suggests a role for epigenetic mechanisms in mediating susceptibility to the toxic effects of lead and cadmium exposure.

### Conclusions

Extreme QTL mapping using *D*. *melanogaster* is an effective approach for the identification of allelic variants associated with variation in resistance to environmental toxins. Identification of human orthologs of Drosophila candidate genes previously associated with variation in heavy metal toxicity validates the Drosophila model as a powerful translational gene discovery system. Orthologous human networks based on networks of Drosophila candidate genes not only provide functional contexts for known human toxicity targets, but can also identify additional candidate susceptibility genes—and therapeutic targets—based on the “guilt-by-association” principle. Thus, the Drosophila model can serve as a gene discovery system to generate candidate networks of human genes that can be tested for variation in susceptibility to heavy metal exposure.

## Materials and methods

### Drosophila lines

We generated an advanced intercross population (AIP) through a round-robin cross design of 37 inbred wild-derived *D*. *melanogaster* lines from the DGRP, followed by over 60 generations of random mating. The 37 founding inbred lines are minimally related, maximally homozygous, have standard karyotypes for all common polymorphic inversions, and are not infected with *Wolbachia*. To minimize genetic drift, the AIP is maintained in 8 bottles at large population sizes and at each generation randomly selected flies of both sexes are combined in new bottles to start the next generation [83].

For functional analyses, we used both *Mi{ET1}* mutants in the *w*^*1118*^ genetic background and *UAS-RNAi* lines with no predicted off-target effects from the Vienna Drosophila Resource Center (VDRC) collection [84]. We crossed each *UAS-RNAi* line and its corresponding control (GD: stock v60000; *w*^*1118*^, KK: stock v60100, *y*,*w*^*1118*^*; P{attP*,*y*^*+*^,*w*^*3`*^*}*) with a weak ubiquitin driver, *Ubi-GAL4* [85], and the F1 offspring were tested for resistance to lead or cadmium exposure.

All flies were maintained on molasses cornmeal-agar medium unless otherwise specified at an ambient temperature of 25°C, 70% humidity and a 12h:12h light-dark cycle.

### Dose-response curves

To establish optimally discriminating concentrations for effects of heavy metal exposure, we generated dose-response curves for both the AIP and the *Mi{ET1}* and *UAS-RNAi* control lines. We collected 3–7 day-old mated flies reared under standard conditions, and placed 5 single sex flies in each vial containing Carolina Formula 4–24^**®**^ potato food supplemented with lead (IV) tetraacetate (Pb(C_2_H_3_O_2_)_4_) or cadmium chloride (CdCl_2_). For lead acetate we tested concentrations of 0 mM, 0.5 mM, 5 mM, 25 mM, 50 mM and 100 mM for the AIP; and 0 mM, 75 mM, 100 mM, 125 mM and 150 mM for the *Mi{ET1}* and *UAS-RNAi* control lines. For cadmium chloride, we tested 0 mM, 25 mM, 50 mM, 100 mM, and 250 mM for the AIP; and 0 mM, 5 mM, 15 mM, 25 mM, 50 mM, and 75 mM for the *Mi{ET1}* and *UAS-RNAi* control lines. For each concentration, we reared five replicate samples of five flies for each sex separately, and counted the number of surviving flies daily.

### Heavy metal resistance

To perform extreme QTL analyses, we selected individuals with extreme resistance to either lead acetate or cadmium chloride and the same number of randomly selected flies. We collected 3–7 day-old mated flies reared under standard conditions, and placed 5 flies of the same sex in vials with either lead or cadmium supplemented medium (3 grams of Carolina Formula 4–24^**®**^ potato food, and 4ml of 75 mM lead acetate solution or 4ml of 25 mM cadmium chloride solution). Experiments were set up in blocks of 250 males and 250 females. Flies were counted each day until only ~10% remained alive; these survivors were flash frozen on dry ice for subsequent DNA extraction. In total, we collected three independent pools of ~100 resistant flies and three pools of 100 random control flies for each sex for each heavy metal treatment.

For functional analyses, we counted the number of surviving flies on the fifth day after flies were placed on medium supplemented with 150 mM lead acetate or 25 mM cadmium chloride. Mutant lines and RNAi lines were always measured contemporaneously with their corresponding control genotype. We measured 15–20 replicates per sex of each genotype, 5 flies per replicate. We performed statistical analyses for each mutant or RNAi line and the corresponding control line separately, using an ANOVA model: *Y* = *μ* + *L* + *S* + *T* + *L* × *S* + *S* × *T* + *L* × *T* + *S* × *T* + *L* × *S* × *T* + *ε*, where *μ* is the overall mean, *L* designates the line effect (mutant vs.control), *S* designates the sex effect (males vs. females), *T* designates the effect of treatment (lead vs. cadmium exposure) and *ε* is the residual variance. Significance of the line, line by sex, line by metal, and line by sex by metal terms all indicate an effect of the mutation or RNAi-suppression of expression on sensitivity to lead and/or cadmium. We also performed ANOVA for each metal and sex separately using the reduced model: *Y* = *μ* + *L* + *ε*. Pooled standard errors were calculated as: $\sqrt{\frac{S_{m}^{2}\left(n_{m}-1\right)+S_{c}^{2}\left(n_{c}-1\right)}{\left(n_{m}-1\right)+\left(n_{c}-1\right)}}*\sqrt{\frac{1}{n_{m}}+\frac{1}{n_{c}}}$, where $S_{m}^{2}$ is the phenotypic variance of the specific mutational line, $S_{c}^{2}$ is the phenotypic variance of the corresponding control line, *n*_*m*_ is the sample size of the same mutational line and *n*_*c*_ is the sample size of the corresponding control line.

To validate RNAi knockdown efficiency of candidate hub genes with human orthologs, we collected 5–7 day old F1 offspring from 12 available RNAi lines and a weak *Ubiquitin-Gal4* driver, as well as F1 offspring from their corresponding controls crossed to the same driver. We extracted total RNA from each line with 2 replicates of 10 flies, sexes separately. Total RNA was quantified by Nano Drop^®^ and normalized to equal concentrations before conversion to cDNA. We performed real-time PCR (BioRad) with 2 technical replicates from each sample and performed *t*-tests on ΔCt values between knockdown lines and their controls.

### DNA sequencing

We homogenized and extracted DNA from pools of 100 flies from either resistant or control samples. We fragmented samples of genomic DNA using a Covaris S220 sonicator to an average size of 300bp and prepared barcoded libraries using NEXTflex^™^ DNA Barcodes (Bioo Scientific, Inc.) according to an Illumina TrueSeq compatible protocol. Libraries were quantified using Qubit dsDNA HS Kits (Life Technologies, Inc.) and a Bioanalyzer (Agilent Technologies, Inc.) to calculate molarity. Libraries were then diluted to equal molarity and re-quantified, and all 24 barcoded samples were pooled. Pooled library samples were quantified again to calculate final molarity and then denatured and diluted to 16pM. They were clustered on an Illumina cBot and sequenced on 8 Illumina Hiseq2500 lanes using 125 bp paired-end v4 chemistry to reach a sequencing depth of ~1X per fly.

### Extreme QTL mapping

We aligned Illumina sequence reads to the Dmel 5.13 reference genome with the Burrows-Wheeler Aligner (BWA) [86] using default parameters and analyzed the aligned sequences using an established pipeline [50]. Briefly, we used GATK software [87] to locally realign regions around indels, remove duplicate sequence reads, and recalibrate base quality scores. We performed local realignment on the BAM files of individual replicates for each heavy metal. Alignments were piled up at each base position in the genome by SAMTools [88]. We filtered SNPs according to the following criteria: alleles were present in the founding strains; coverage of Q13 bases was between 20 and 1,500; at least 80% of the coverage was at least Q13; the two most frequent alleles constituted at least 95% of all observed alleles; minor alleles were present by at least 2.5% in one of the pools; the Chernoff bound of the *P* value for the null hypothesis that the observed minor alleles were caused by sequencing error [89] was smaller than 10^−5^; and strand bias was not significant (*P* > 10^−5^) in both resistant and control pools. Allele frequencies were estimated by calculating the proportion of reads supporting the alleles. We tested for differences in allele frequencies between the resistant and control pools by computing $Z=\left(p_{1}-p_{2}\right)/\sqrt{p_{0}\left(1-p_{0}\right)\left(\frac{1}{n}+\frac{1}{d_{1}}+\frac{1}{d_{2}}\right)}$, where *p*_*1*_ and *p*_*2*_ are the estimated allele frequencies in the resistant and control pools, respectively; *p*_*0*_ is the allele frequency under the null hypothesis: *p*_*1*_ *= p*_*2*_ estimated from the average of *p*_*1*_ and *p*_*2*_; *n* is the number of flies (*n* = 300) in the pools; and *d*_*1*_ and *d*_*2*_ are the sequencing depths for the resistant and control pools, respectively. *P* values were obtained by comparing the *Z* statistics to the standard normal distribution. We considered differences in allele frequency with a False Discovery Rate (FDR) of FDR<0.05 to be significant.

### Gene annotation and network analysis

We performed gene ontology enrichment analysis using flymine.org, for genes with differentially segregating SNPs at FDR<0.05, and constructed gene networks with known physical and genetic interactions using esyN through flymine.org [52, 90]. Networks of human orthologs of the same genes were constructed using humanmine.org [91].

## Supporting information

S1 Fig Volcano plots of differentially segregating allelic variants associated with adult resistance to lead and cadmium exposure. (PDF)

S2 Fig Venn diagrams showing overlap of SNPs and genes associated with adult susceptibility to lead (Pb) and cadmium (Cd) exposure for each sex (M, males; F, females). (PDF)

S3 Fig Dose-response curves for survival of adult flies of genetic background control strains of *Mi{ET1}* mutants and KK and GD *UAS-RNAi* lines reared on lead acetate and cadmium chloride-supplemented media. The plots represent the average number of dead flies after exposure to lead or cadmium for five days.
(PDF)

S4 Fig Networks of human orthologs of Drosophila candidate genes associated with susceptibility for lead and cadmium. (A) Network of human orthologs of Drosophila candidate genes associated with both female and male resistance to lead exposure. (B) Human orthologs of Drosophila candidate genes associated with both female and male resistance to cadmium exposure. (C) Interaction diagrams of human orthologs of Drosophila candidate genes associated with male resistance to both lead and cadmium exposure. (D) Network and interaction diagrams of human orthologs of Drosophila candidate genes associated with female resistance to both lead and cadmium exposure. Orange edges indicate physical interactions and green edges indicate genetic interactions.
(PDF)

S5 Fig Expression changes of candidate genes in RNAi knockdown lines. The bar graphs represent fold changes of gene expression between RNAi knockdown lines and their corresponding controls using real-time PCR. Red bars indicate females and blue bars indicate males. *: *P*<0.05, **: *P*<0.01, ***: *P*<0.001.
(PDF)

S1 Table Extreme QTL analysis for adult resistance to lead exposure. (XLSX)

S2 Table Extreme QTL analysis for adult resistance to cadmium exposure. (XLSX)

S3 Table Fly stocks used to perform functional analyses for validation of candidate genes. (XLSX)

S4 Table Full model ANOVA for validation of candidate genes using (A) *Mi{ET1}* mutants and (B) *UAS-RNAi* knockdown lines. (XLSX)

S5 Table Reduced model ANOVA for validation of candidate genes using (A) *Mi{ET1}* mutants and (B) *UAS-RNAi* knockdown lines. (XLSX)

S6 Table Gene ontology (GO) analysis for (A) genes associated with resistance to lead and genes associated with resistance to cadmium combined, (B) genes associated with resistance to lead, (C) genes associated with cadmium. (XLSX)

S7 Table Analyses in which Drosophila candidate genes associated with resistance to lead and/or cadmium were identified. (XLSX)

S8 Table Gene ontology analyses for genes associated with (A) lead resistance in both sexes, (B) cadmium resistance in both sexes, (C) lead and cadmium resistance in females and (D) lead and cadmium resistance in males. (XLSX)

S9 Table Gene ontology (GO) analysis for human orthologs of all Drosophila candidate genes associated with resistance to (A) lead and cadmium, (B) lead in females, (C) lead in males, (D) cadmium in females and (E) cadmium in males. (XLSX)

S10 Table Gene ontology analyses for human orthologs of Drosophila candidate genes associated with resistance to (A) lead in both sexes, (B) cadmium in both sexes, (C) lead and cadmium in females and (D) lead and cadmium in males. (XLSX)

S11 Table ANOVA for validation of Drosophila orthologs of human hub genes using RNAi knockdown lines. (XLSX)
