# Supplementary figures and images for: A Drosophila model for toxicogenomics: Genetic variation in susceptibility to heavy metal exposure
Source: PLoS Genet. 2017 Jul 21;13(7):e1006907. doi: 10.1371/journal.pgen.1006907 (PMC5544243; doi:10.1371/journal.pgen.1006907)

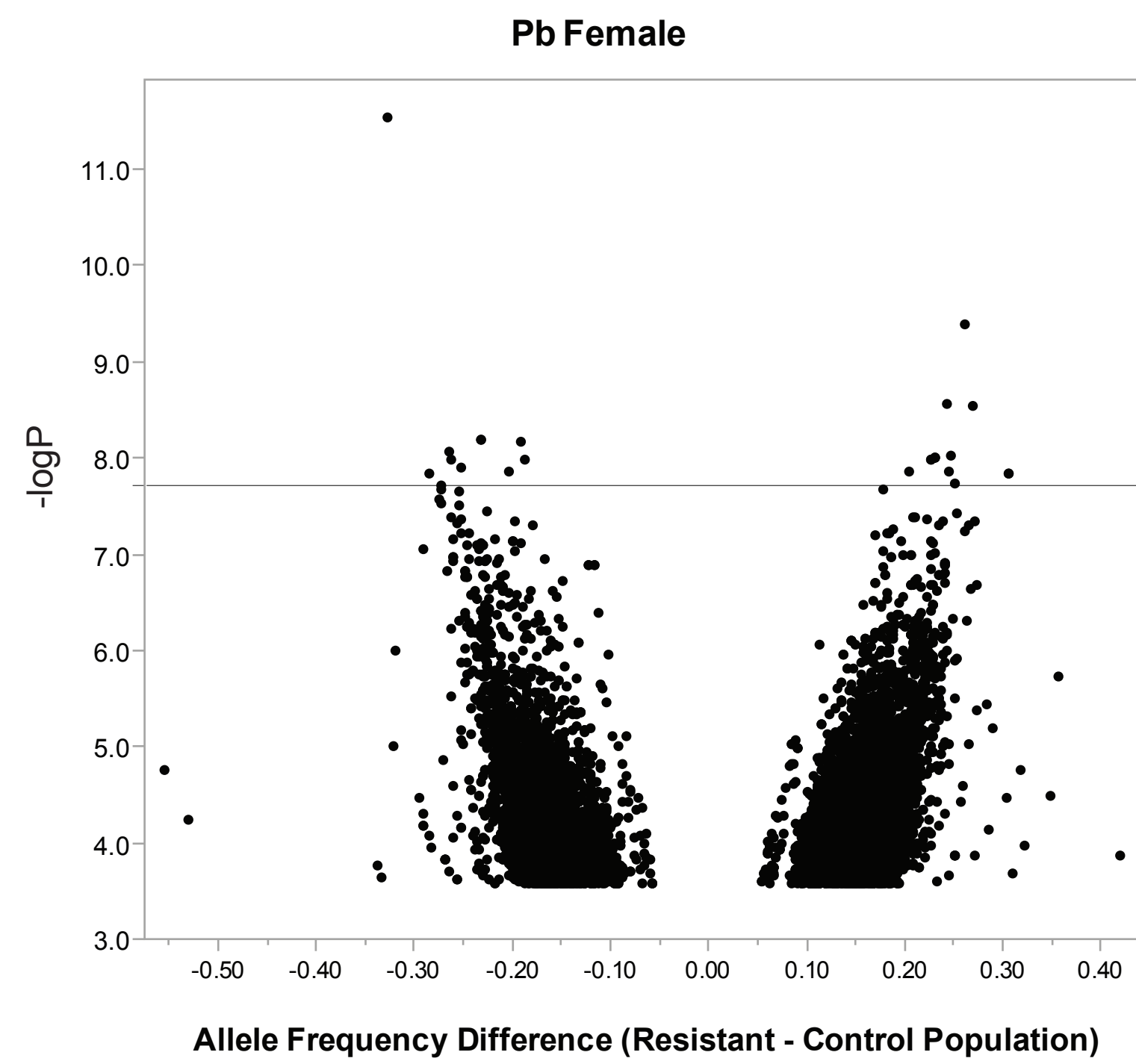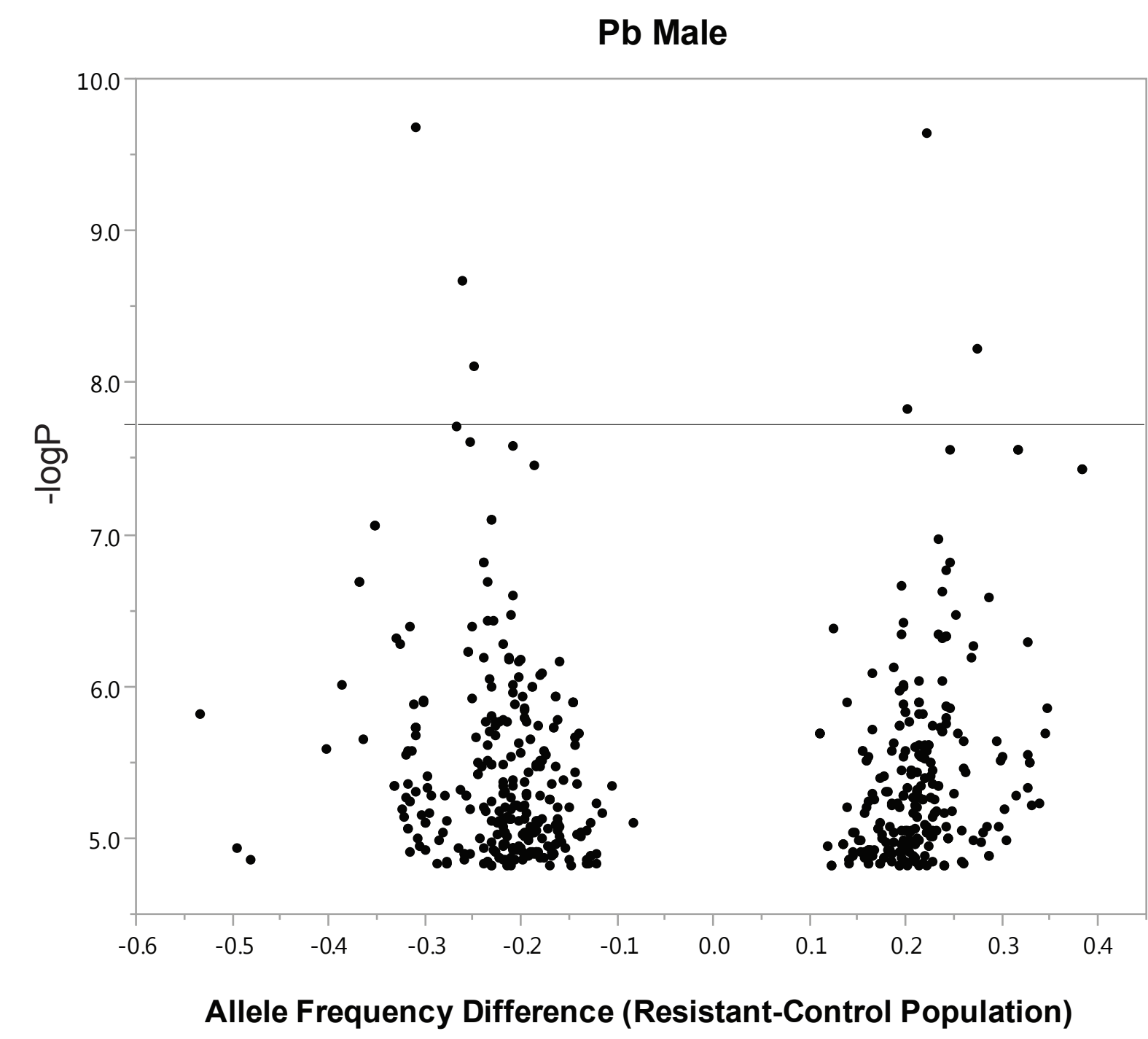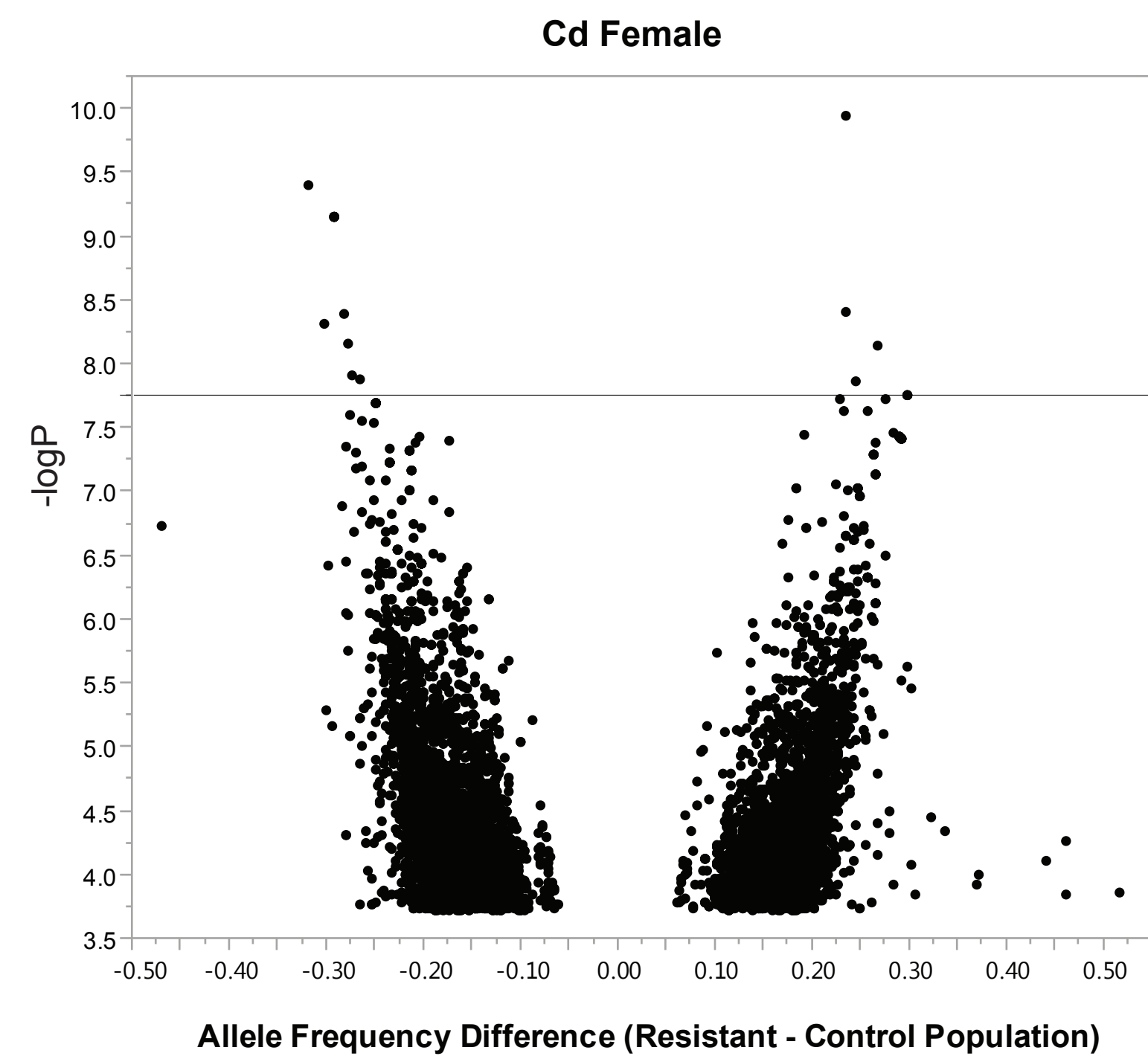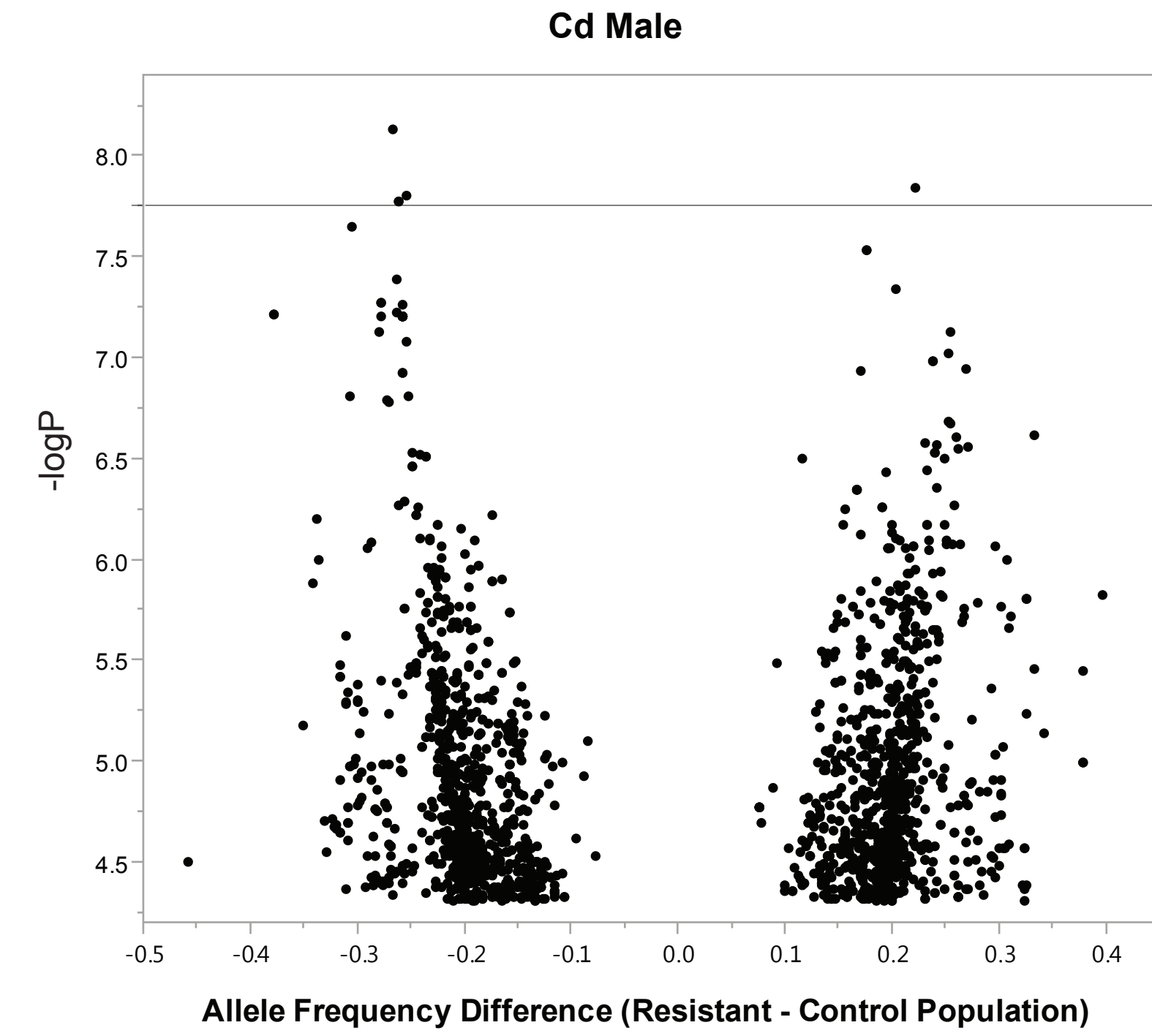

Supplement: S1 Fig — (PDF) [file pgen.1006907.s001.pdf]

SNP Overlap

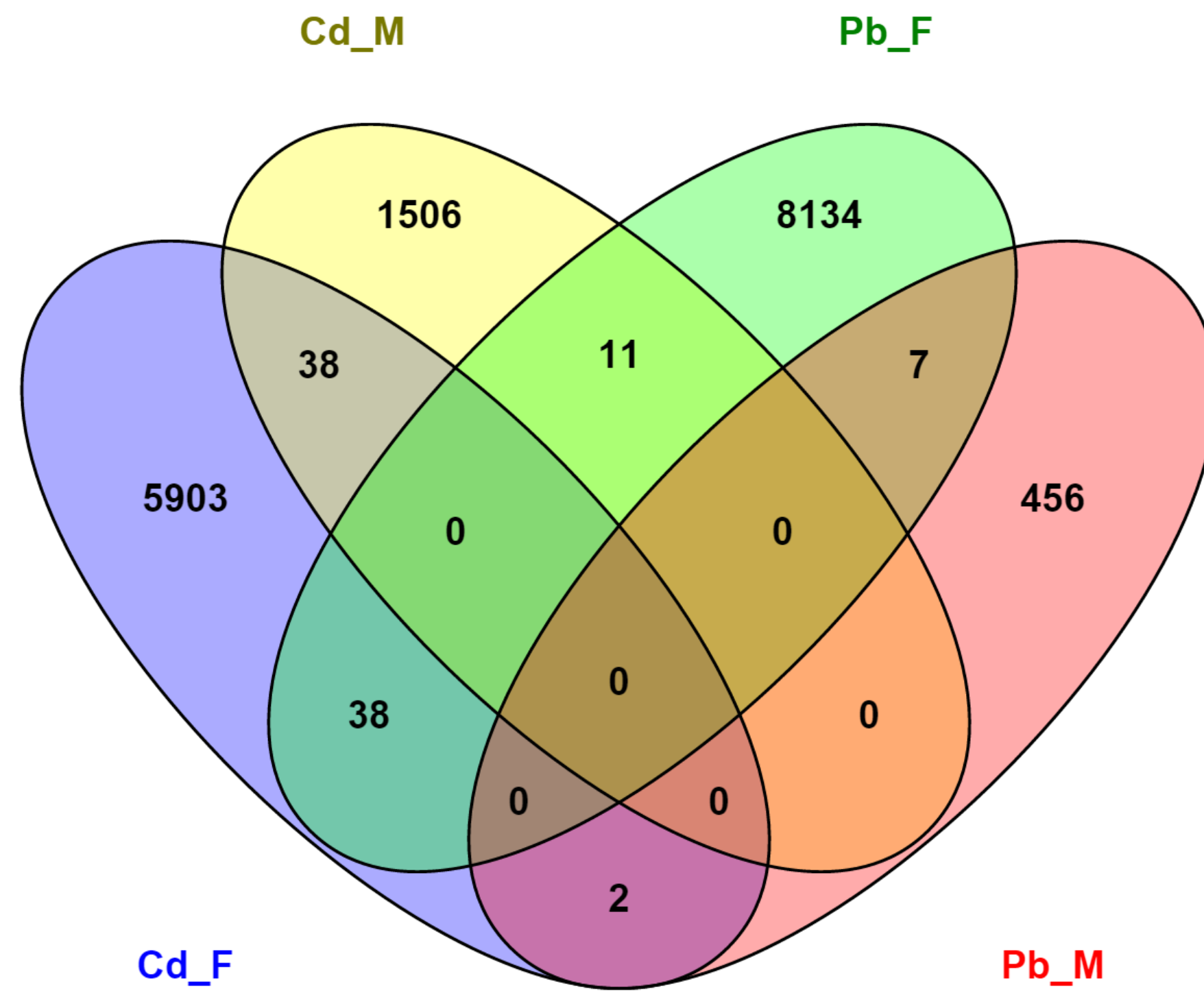

Gene Overlap

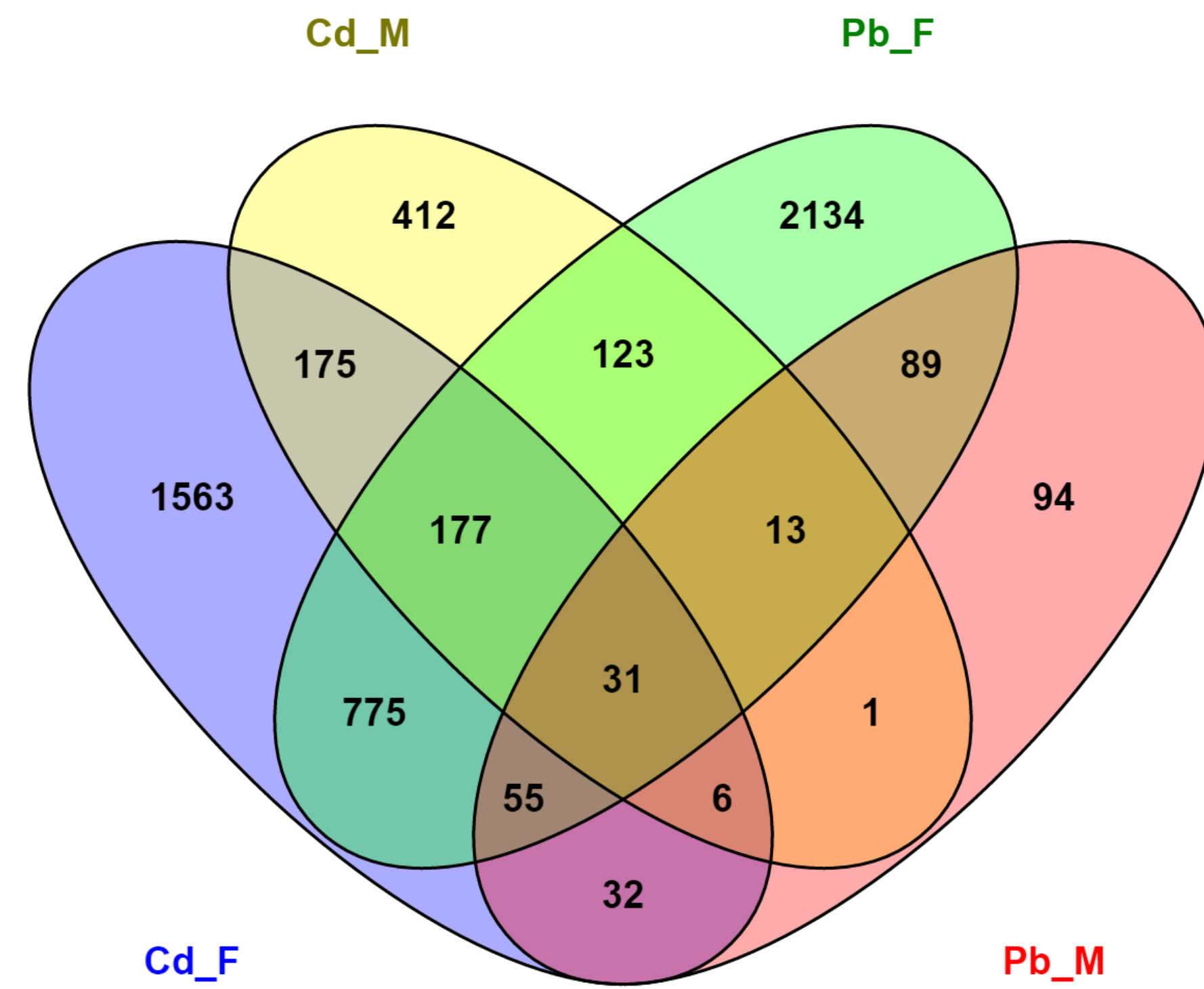

Supplement: S2 Fig — (PDF) [file pgen.1006907.s002.pdf]

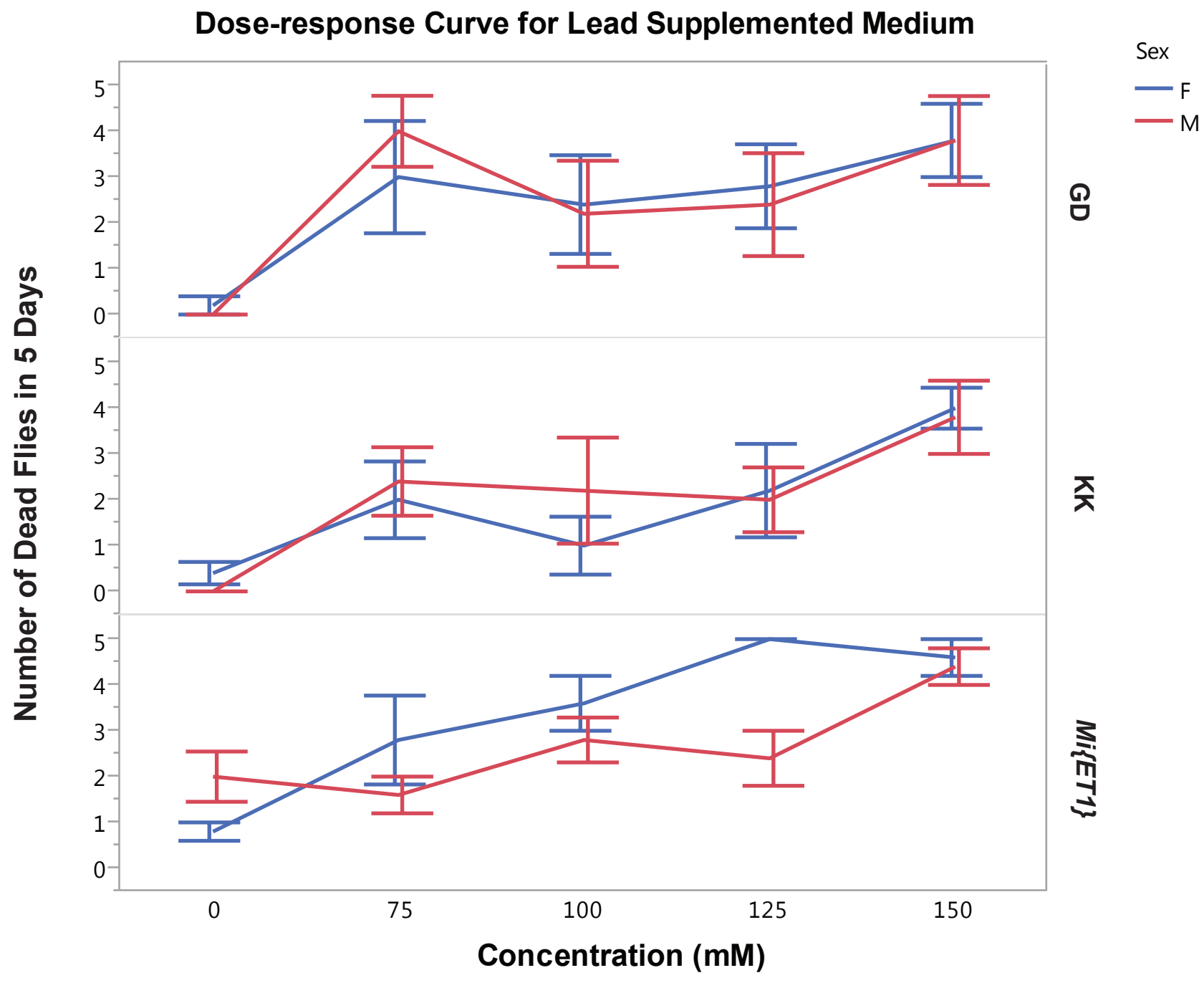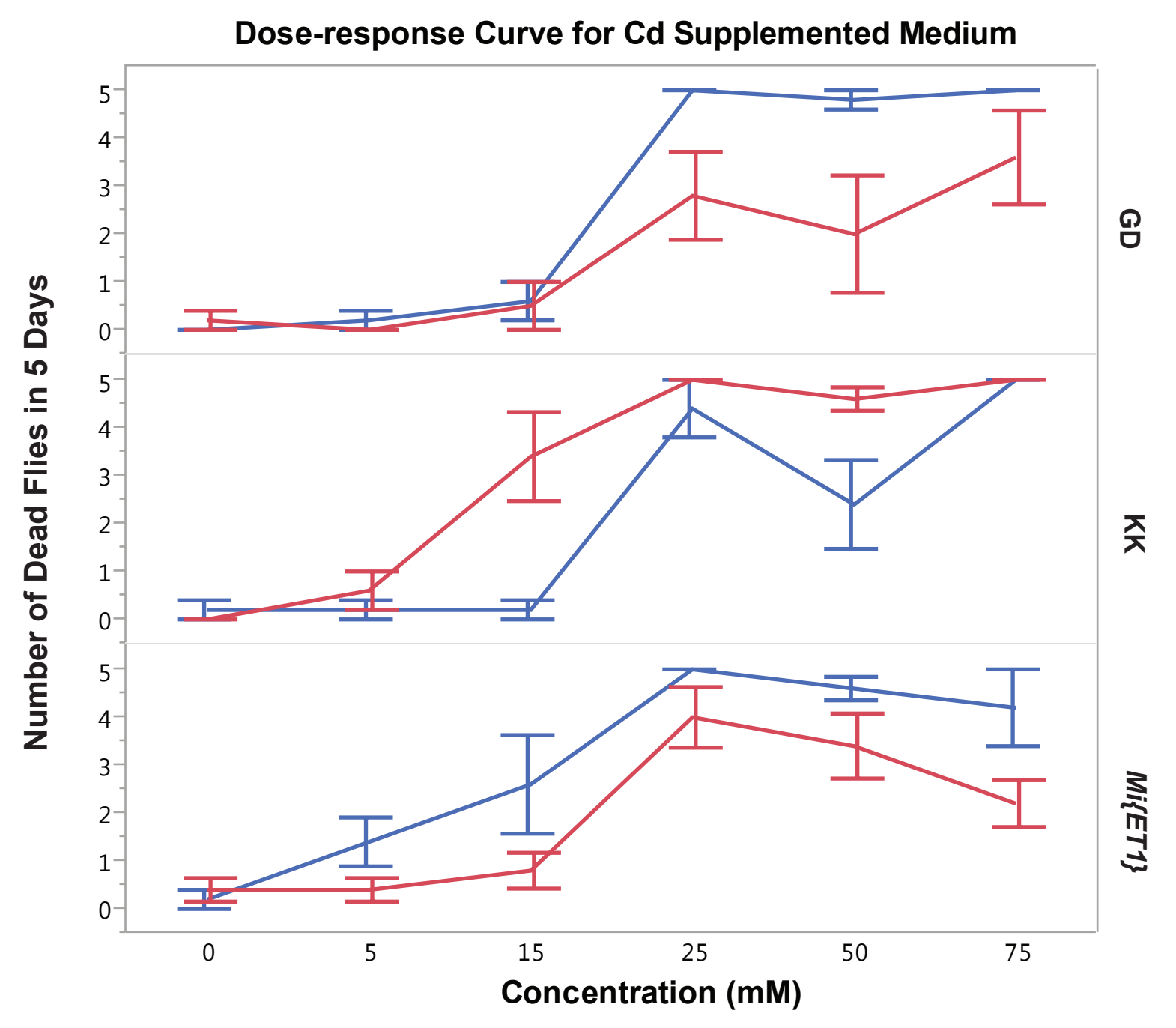

Supplement: S3 Fig — The plots represent the average number of dead flies after exposure to lead or cadmium for five days. (PDF) [file pgen.1006907.s003.pdf]

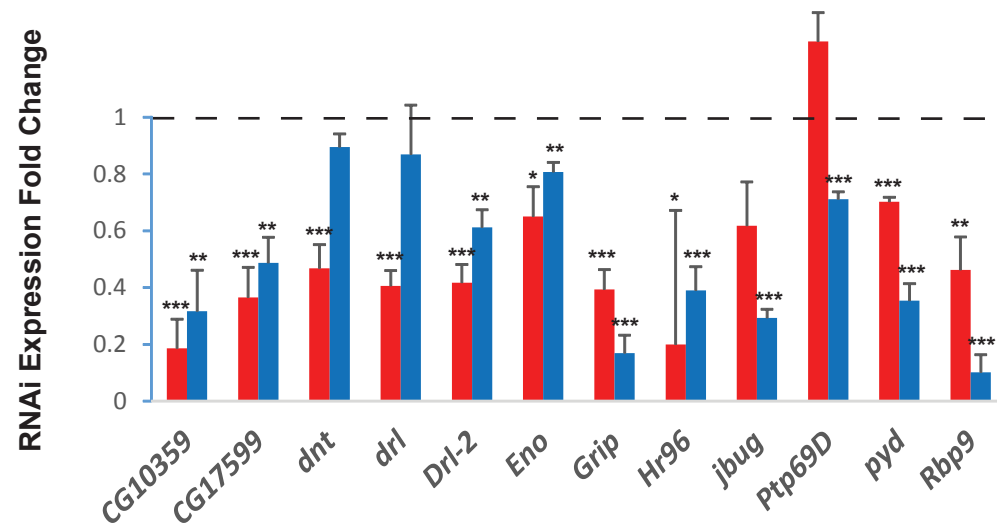

Supplement: S5 Fig — The bar graphs represent fold changes of gene expression between RNAi knockdown lines and their corresponding controls using real-time PCR. Red bars indicate females and blue bars indicate males. *: P<0.05, **: P<0.01, ***: P<0.001. (PDF) [file pgen.1006907.s005.pdf]
