# Supplementary material for: A Drosophila model for toxicogenomics: Genetic variation in susceptibility to heavy metal exposure
Source: PLoS Genet. 2017 Jul 21;13(7):e1006907. doi: 10.1371/journal.pgen.1006907 (PMC5544243; doi:10.1371/journal.pgen.1006907)
Supplement: S4 Fig — (A) Network of human orthologs of Drosophila candidate genes associated with both female and male resistance to lead exposure. (B) Human orthologs of Drosophila candidate genes associated with both female and male resistance to cadmium exposure. (C) Interaction diagrams of human orthologs of Drosophila candidate genes associated with male resistance to both lead and cadmium exposure. (D) Network and interaction diagrams of human orthologs of Drosophila candidate genes associated with female resistance to both lead and cadmium exposure. Orange edges indicate physical interactions and green edges indicate genetic interactions. (PDF) [file pgen.1006907.s004.pdf]

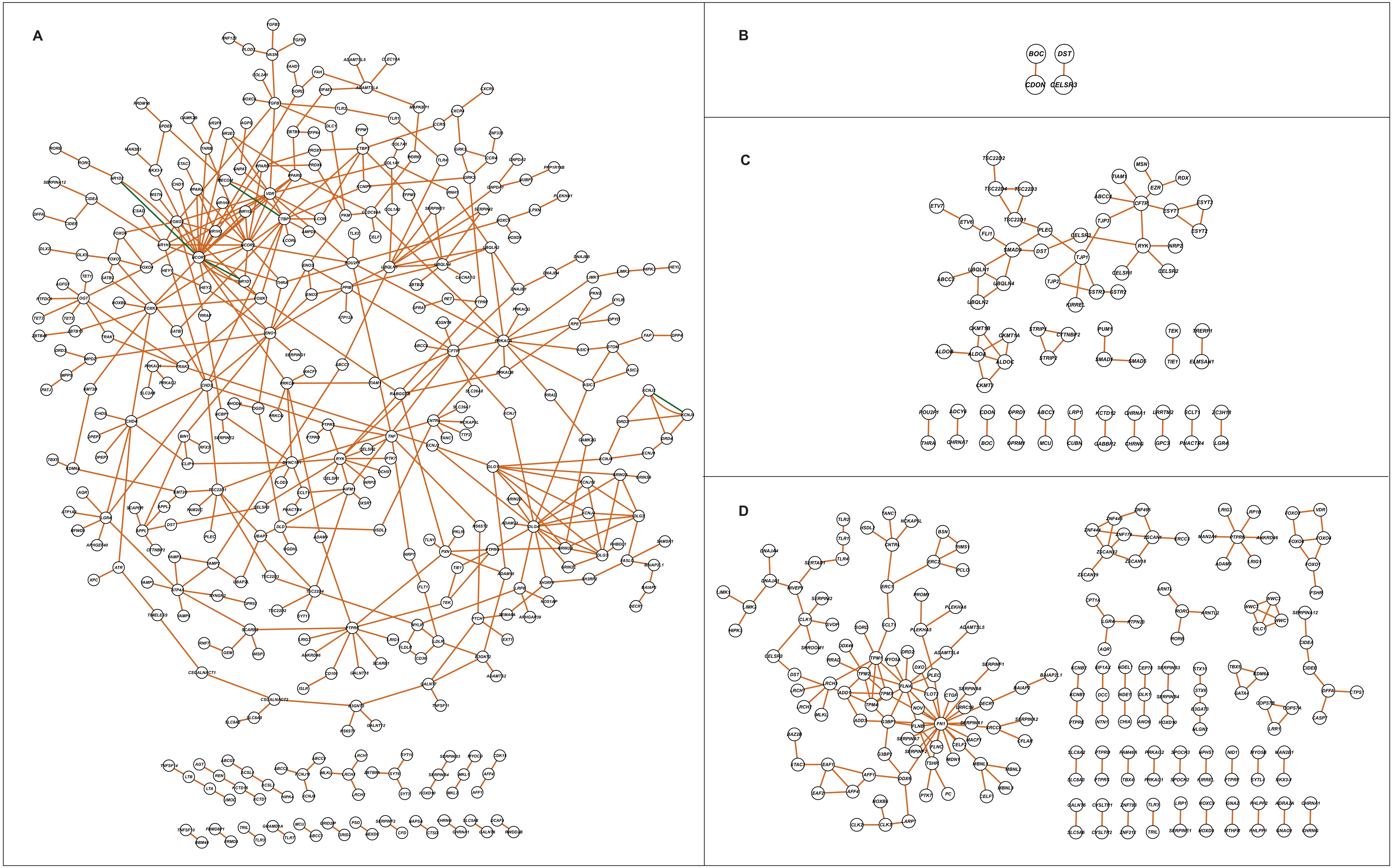
A
B
BOC
DST
CDON
CELSR3
C
D
